# Development of Axon-Target Specificity of Ponto-Cerebellar Afferents

**DOI:** 10.1371/journal.pbio.1001013

**Published:** 2011-02-08

**Authors:** Anna Kalinovsky, Fatiha Boukhtouche, Richard Blazeski, Caroline Bornmann, Noboru Suzuki, Carol A. Mason, Peter Scheiffele

**Affiliations:** 1 Department of Physiology & Cellular Biophysics and Department of Neuroscience, Columbia University, New York, New York, United States of America; 2 Biozentrum, University of Basel, Basel, Switzerland; 3 Department of Pathology & Cell Biology and Department of Neuroscience and Ophthalmology, Columbia University, New York, New York, United States of America; 4 Mie University Life Science Research Center of Animal Genomics, Functional Genomics Institute, Japan; The Scripps Research Institute, United States of America

## Abstract

The function of neuronal networks relies on selective assembly of synaptic connections during development. We examined how synaptic specificity emerges in the pontocerebellar projection. Analysis of axon-target interactions with correlated light-electron microscopy revealed that developing pontine mossy fibers elaborate extensive cell-cell contacts and synaptic connections with Purkinje cells, an inappropriate target. Subsequently, mossy fiber–Purkinje cell connections are eliminated resulting in granule cell-specific mossy fiber connectivity as observed in mature cerebellar circuits. Formation of mossy fiber-Purkinje cell contacts is negatively regulated by Purkinje cell-derived BMP4. BMP4 limits mossy fiber growth in vitro and Purkinje cell-specific ablation of BMP4 in mice results in exuberant mossy fiber–Purkinje cell interactions. These findings demonstrate that synaptic specificity in the pontocerebellar projection is achieved through a stepwise mechanism that entails transient innervation of Purkinje cells, followed by synapse elimination. Moreover, this work establishes BMP4 as a retrograde signal that regulates the axon-target interactions during development.

## Author Summary

Brain functions rely on highly selective neuronal networks which are assembled during development. Network assembly involves targeted neuronal growth followed by recognition of the appropriate target cells and selective synapse formation. How neuronal processes select their appropriate target cells from an array of interaction partners is poorly understood. In this study, we have addressed this question for the axons emerging from the pontine gray nucleus, a major brainstem nucleus that relays information between the cortex and the cerebellum, a brain area responsible for the control of skilled movements but also emotional processing. Using advanced microscopy techniques, we find that developing mossy fibers establish synaptic contacts rather promiscuously, and elaborate extensive synapses with Purkinje cells, an inappropriate target. These contacts are subsequently eliminated, and proper synaptic connectivity is then restricted to granule and Golgi neurons. We identify bone morphogenetic protein 4 (BMP4) as a regulator of these inappropriate mossy fiber-Purkinje cell contacts. BMP growth factors are best known for their functions in cell specification during embryonic development, and our results support an additional retrograde signaling function between axons and their target cells in early postnatal stages. In summary, we show that the specificity of the synaptic connections in the ponto-cerebellar circuit emerges through extensive elimination of transient synapses.

## Introduction

The specificity of synaptic connectivity in the central nervous system is a prerequisite for brain function. The neuronal circuits in the vertebrate cerebellum represent a remarkable example of wiring specificity. This was first recognized by Santiago Ramón y Cajal when he chose cerebellar circuits as revealed by the Golgi method for his early studies on brain organization (discussed in [1]). In its simplest form, the cerebellar microcircuit integrates input from two afferent classes—climbing and mossy fibers. Climbing fibers selectively innervate Purkinje cells. By contrast, mossy fiber afferent activity is relayed to Purkinje cells via granule cells in the inner granular layer of the cerebellum (IGL) [2]–[4]. In the IGL, mossy fibers also form synapses on Golgi cells, a class of inhibitory interneurons that provide feed-forward inhibition in the cerebellar circuit. Climbing and mossy fiber information is then integrated in Purkinje cells and transduced via cerebellar efferent projection neurons in the deep cerebellar nuclei. Despite the apparent simplicity of the cerebellar circuit, it is unknown how the specificity of synapse formation emerges during development for each of the principal cerebellar afferent systems. Indeed, the molecular mechanisms regulating synapse specificity for most circuits in the mammalian brain have remained obscure.

Two key steps determining the incipient pattern of synaptic connectivity during development are axon-target contact formation and synaptic differentiation. Ultrastructural reconstruction of mature neuronal circuits suggests that only a subset of contacts differentiates into bona fide synapses [5]. The fraction of actual synapses compared to cellular contacts (potential synapses) has been termed “filling fraction”, with a filling fraction of 1.0 representing a case where all contacts are synaptic structures [6]. In vertebrate and invertebrate systems several attractive and repulsive factors have been identified that contribute to synaptic specificity [7]–[13]. However, pinpointing whether these specificity factors regulate primarily selective contact formation, synaptic differentiation, or both has been challenging, given the limited resolution of light microscopy in assessing direct cellular contacts in vivo. One possibility is that some signaling pathways regulate primarily contact formation, whereas other factors drive the synaptic differentiation process after axon-target contacts are established.

The ponto-cerebellar projection represents an excellent model system to explore mechanisms of synaptic specificity in the mammalian brain [14]. Mossy fiber axons emerging from the basilar pons (PGN) in the ventral brain stem form a major projection to the cerebellar cortex which relays information from sensory and motor cortex. Structurally, mossy fiber afferents exhibit synaptic specificity at two levels: Mossy fiber axons elaborate synapses exclusively with granule and Golgi cells but not Purkinje cells. At the subcellular level, mossy fiber synapses are restricted to the proximal regions of Golgi cells within the IGL but are excluded from the molecular layer where distal Golgi cell dendrites arborize. Cell culture studies indicated that immature granule cells provide a stop-signal for mossy fiber growth [15]. Maturing granule cells, in contrast, contribute positive signals for the differentiation of mossy fiber synapses and the elaboration of mossy fiber glomeruli [16]–[19]. However, it is unknown how contact and synapse specificity emerges for mossy fibers and their granule and Golgi cell targets. Moreover, negative signals that suppress contact of mossy fibers with inappropriate target cells in vivo have not been identified.

Previous anatomical studies indicated that axons with the appearance of mossy fibers do not exhibit strict targeting specificity but form broader projection patterns during early postnatal stages [20]–[22]. Fibers with mixed mossy fiber and climbing fiber morphology (“combination fibers”) were observed to contact Purkinje cells during development though the extent of these interactions remained unknown. A developmental gene expression analysis in pontine nuclei revealed distinct transcriptional programs for axonal growth and synaptic differentiation of pontine mossy fibers [23]. Surprisingly, the termination of the axonal growth program did not require the presence of granule cells in the cerebellar cortex but was perturbed in mutant mice with Purkinje cell degeneration accompanied by granule cell death [23]. In combination, these studies raised the question of whether Purkinje cells may provide signals for the development of mossy fiber projections.

To define the cellular nature of mossy fiber–Purkinje cell interactions and the rearrangements resulting in the specific synaptic wiring pattern, we undertook a systematic analysis of axon-target interactions in the mouse ponto-cerebellar system. Using correlated light-electron microscopy analysis we quantitatively mapped physical contacts and synaptic structures formed between identified pontine mossy fibers and Purkinje cells. Using this methodology, we observed extensive transient mossy fiber contacts and synapses on Purkinje cells that are subsequently eliminated. Patterning molecules such as WNTs, FGFs, and BMPs have been shown to exert novel neuronal signaling functions at the *Drosophila* neuromuscular junction and in the mammalian central nervous system [24]–[26]. Of this class of molecules, the BMPs have been extensively studied as retrograde signals at the *Drosophila* neuromuscular junction [27]–[32] and as trophic factors in mammalian neurons [33]–[35]. However, their signaling functions in vertebrate axon–target interactions have not been determined. We explored a role for BMP signaling in mossy fiber transient target interactions in the developing cerebellum. Using expression analysis, in vitro assays, and conditional knock-out mice we identify BMP4 as Purkinje cell-derived signal that specifically controls mossy fiber–target contact selectivity during development.

## Results

### Establishment of Mossy Fiber Target Specificity during Development

**Figure 1:**
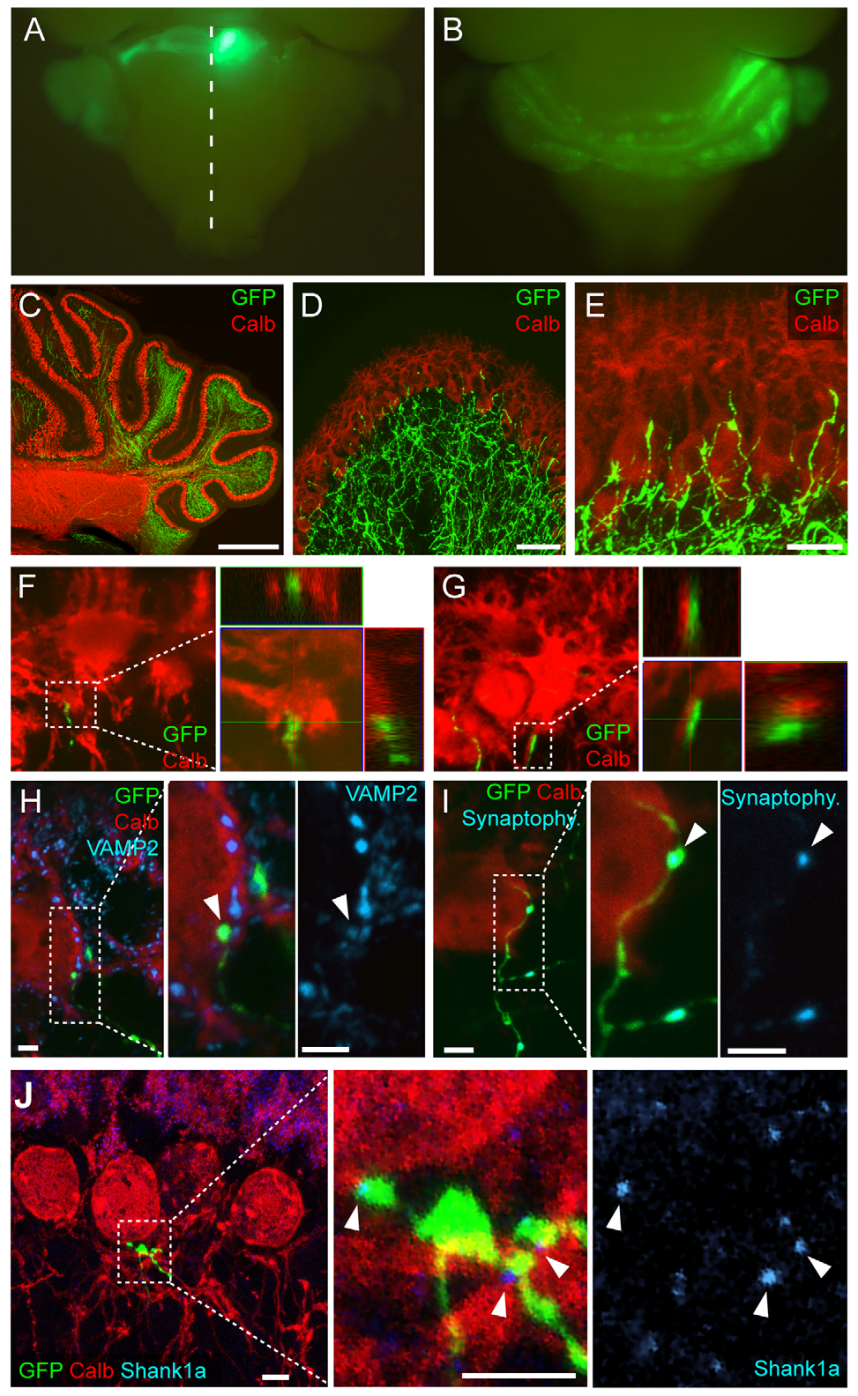
Mossy fiber–Purkinje cell contacts in the developing mouse cerebellum. (A) Pontine mossy fibers were selectively labeled by *in utero* electroporation at E14.5. Ventral view of a pup (P7) shows highly selective marking of cells in the pontine gray nucleus (see Figure S1 and Methods). The midline is indicated as a dotted line. (B) Dorsal view of cerebellum with mossy fiber axon projections in a P7 electroporated mouse. (C–E) GFP-labeled mossy fiber axons (green) projecting to the mediolateral cerebellum at P7. Purkinje cells are marked with anti-calbindin antibodies (Calb) in red. (F,G) Left panels: High-magnification views of mossy fiber–Purkinje cell contacts at P7 formed on the Purkinje cell soma (F) and the Purkinje cell axon (G). Rights panels show an orthogonal projection of a 0.45 µm thick section at the site of contact, showing the apposition of the cellular markers Calb (red) and GFP (green). (H, I) Mossy fiber–Purkinje cell contacts accumulate presynaptic markers. The EGFP-positive varicosities contain endogenous synaptic vesicle protein VAMP2 (H, blue) or synaptophysin-fluorescent protein fusion introduced by co-electroporation (I, blue) with EGFP. (J) In Thy1.2-EGFP-O mice a population of mossy fibers from multiple precerebellar nuclei is labeled at P14 (for details see Figure S1). At a subset of mossy fiber–Purkinje cell contacts the postsynaptic density marker Shank1a (blue) is concentrated. Scale bars: (C) 500 µm; (D) 50 µm; (E) 20 µm; (H,I) 5 µm; (J) 10 µm.

To examine the emergence of synaptic specificity of ponto-cerebellar mossy fibers we adopted an *in utero* electroporation approach [36],[37]. Pontine precursor cells are selectively electroporated by injection of DNA constructs into the 4^th^ ventricle at embryonic day 14.5 (Figure S1). Following differentiation and migration, these cells settle into the pontine gray nucleus (Figure 1A,B) [36],[37]. Pontine axons labeled by electroporation of an EGFP expression plasmid project to the cerebellar cortex assume typical mossy fiber morphology and are restricted to the IGL at postnatal day 21 (P21), consistent with the selective elaboration of mossy fiber–granule cells synapses in the adult cerebellar circuitry (Figure S1). To examine how this target specificity emerges, we examined earlier developmental time points. At P7, we identified a significant number of GFP-positive mossy fiber extensions projecting beyond the IGL into the Purkinje cell layer (PCL) (Figure 1C–E). Using three-dimensional analysis of high resolution confocal stacks, mossy fiber varicosities were found in direct proximity with calbindin-positive Purkinje cell somata and axons, suggesting direct mossy fiber–Purkinje cell contacts (Figure 1F,G). These contacts contained synaptic markers, as they concentrated the endogenous synaptic vesicle protein VAMP2 or a synaptophysin-fluorescent protein fusion that was introduced by electroporation into the pontine projection neurons (Figure 1H,I).

Mossy fibers emerge not only from the PGN but multiple pre-cerebellar nuclei. The elaboration of mossy fiber–Purkinje contacts was not unique to PGN-derived mossy fibers as it was also observed in GFP-O transgenic mice where multiple other mossy fiber populations are marked by EGFP (Figure S1) [38]. Some of these contacts concentrated the postsynaptic scaffolding protein Shank1a (Shank1a-positive: 16% of somatic contacts, *n* = 50 contacts; 44% of contacts with proximal PC axon segments, *n* = 167; Figure 1J). In sum, these findings suggest that mossy fiber afferents establish transient synapse-like contacts with Purkinje cells during postnatal development.

### Correlated Light-Electron Microscopy Reveals Transient Mossy Fiber–Purkinje Cell Synapses

**Figure 2:**
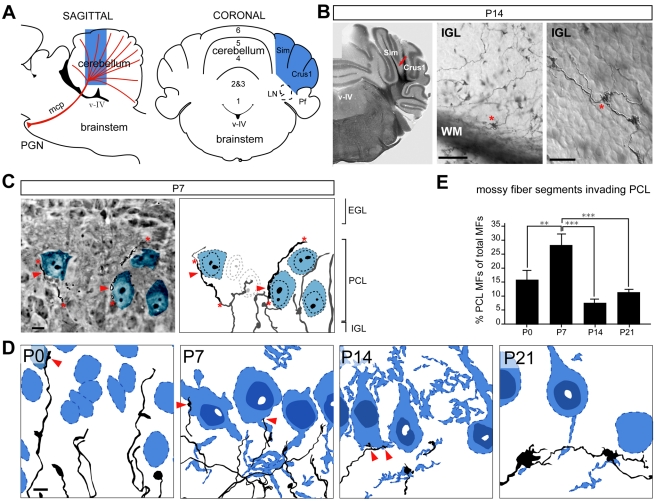
Transient innervation of pontine mossy fibers into Purkinje cell territory during postnatal development. (A) Schematic diagram showing position of DiI crystals insertion in the PGN (red triangle). Cerebellar hemispheres in which DiI was photoxidized forming dark precipitate are highlighted in blue. (B) Photoxidized DiI-labeled mossy fibers visualized in 50 µm coronal sections at P14 show typical mossy fiber morphologies. Red arrow in the left panel points at labeled mossy fiber axons traversing white matter (WM). Middle panel shows mossy fibers exiting the axon tract and arborizing in the IGL form multiple irregularly shaped varicosities (rosettes). Right panel outlines unlabeled cells in the cerebellar cortex, and their cellular relationships with labeled mossy fibers. Asterisks highlight examples of immature rosettes in the IGL characteristic during the second postnatal week [20]. Scale bars: middle panel 50 µm, right panel 10 µm. EGL, external granule cell layer; PCL, Purkinje cell layer; IGL, inner granule cell layer; WM, white matter; Sim, simplex lobule; mcp, medial cerebellar peduncle; v–IV, fourth ventricle; Pf, paraflocculus. (C) High magnification photograph of 7 µm semithin section from cerebellar cortex at P7, next to *camera lucida* drawing reconstructing mossy fiber axonal arborizations and their cellular relationships with Purkinje cell somata from 50 µm thick section of the same area. Several Purkinje cell somata are highlighted in blue. Since only a single plane of focus can be visualized in the photomicrograph, mossy fibers appear as discontinuous segments (delineated by asterisks). Reconstruction from multiple focal planes using *camera lucida* allows us to visualize continuous axonal arbors. Red arrowheads point at putative contacts on Purkinje cell somata. Mossy fiber segments and Purkinje cells that are not on the same focal plane as the photograph are grayed out in the *camera lucida* drawing. Scale bar: 10 µm. (D) C*amera lucida* drawings of the upper IGL and the PCL of DiI-calbindin double-labeled 50 µm sections from P0, P7, P14, and P21 time points. Purkinje cells are shown in blue. Red arrows point at putative contacts between mossy fibers and Purkinje cell somata. Scale bar: 10 µm. (E) Quantitative assessment of mossy fiber invasion into Purkinje cell territory from *camera lucida* drawings at P0, P7, P14, and P21 time points. Drawings encompass all of the mossy fiber segments and Purkinje cell outlines reconstructed from an area 175 µm horizontal×120 µm vertical×approximately 30 µm deep (thickness of one Purkinje cell soma), oriented parallel to the PCL, and recorded with a 100× objective. The percentage of all mossy fiber segments that enter the PCL out of all mossy fiber segments was scored (>1,000 segments from >25 areas obtained from 5–9 animals per time point, >3 50 µm sections per animal were analyzed). Statistical significance was determined by Mann-Whitney *t* test. Two-tailed *p* values were used with 95% confidence intervals, ** *p*<0.01, *** *p*<0.001.

In order to visualize the developmental progression of mossy fiber–Purkinje cell contacts and their differentiation into synapses we undertook a systematic light-electron microscopy analysis of PGN-derived mossy fiber axons. Pontine projections were labeled by DiI tracing followed by photoconversion of the dye (Figure 2). Mossy fiber projection patterns and Purkinje cell interactions in the cerebellar hemispheres (Crus1, Crus 2, and Simplex lobules) were quantitatively examined by light microscopy and apparent contacts were subsequently analyzed by electron microscopy. By light microscopy, mossy fiber rosettes and the thin protrusions extending from them are seen in great detail, and somata of Purkinje and granule cells of the cerebellar cortex can be clearly identified by DIC microscopy (Figures 2B,C, S2A–D). To further confirm the identity of the Purkinje cell territory, some sections were additionally labeled with antibodies to calbindin (Figure S2I,J). C*amera lucida* drawings of mossy fiber terminals from 50 µm sections at P0, P7, P14, and P21 revealed that the mature mossy fiber projection pattern emerges from a series of transformations during postnatal development that includes an extensive invasion of and eventual withdrawal from the Purkinje cell territory (Figure 2D,E).

At P0 mossy fibers extend far into the developing cerebellar cortex where Purkinje cells are unevenly distributed and intermixed with migratory and maturing granule cells of the emerging IGL (Figures 2D, S2). At P7, Purkinje cells form a recognizable monolayer above the IGL. However, pontine mossy fibers substantially invade this Purkinje cell territory with close to 30% of all labeled segments in the IGL penetrating into the PCL (Figure 2E,D, see Methods and figure legends for details on quantitative analysis). This invasion of the PCL was significantly reduced at postnatal days 14 and 21, yielding the mature mossy fiber projection pattern. At postnatal days 7 and 14 mossy fibers in close apposition to Purkinje cell somata often exhibited marked varicosities, resembling the presumptive mossy fiber–Purkinje cell synapses identified using the *in utero* electroporation approach (Figure 2C,D).

**Figure 3:**
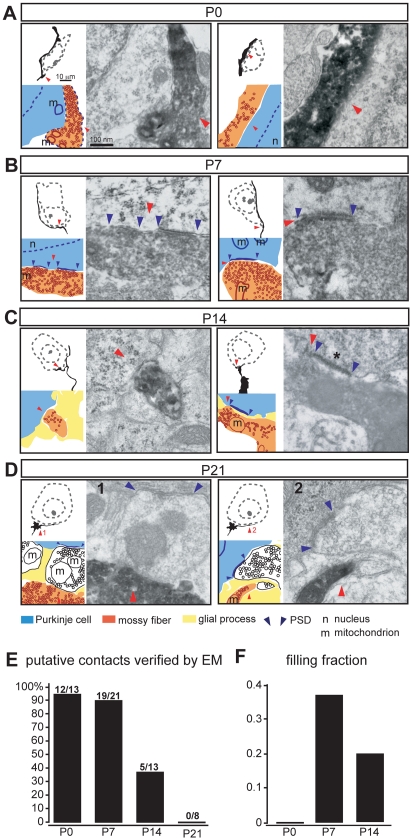
Transient mossy fiber–Purkinje cell contacts and synapses identified by correlated light-electron microscopy. Mossy fiber–Purkinje cell contacts at P0, P7, P14, and P21 time points. Each panel consists of *camera lucida* drawing (top left), corresponding electron micrograph (EM), and a schematic drawn to scale highlighting major ultrastructural elements representing mossy fibers (orange), Purkinje cells (blue), glial processes (yellow), unlabeled terminals (presumably from PC collaterals, white), synaptic vesicles (small circles), and PSDs (demarcated by blue arrowheads). Contacts are highlighted by red arrowheads. Scale bars: *camera lucida* drawings 10 µm, electron micrographs 100 nm. (A) At P0 electron micrographs from the contact region verify direct cellular appositions between the mossy fibers and the Purkinje cells. By light microscopy mossy fibers form extensive putative contacts throughout the entire length of Purkinje cell somata. Even though mossy fiber segments apposed to Purkinje cell somata are densely filled with synaptic-like vesicles, no synaptic specializations can be seen by electron microscopy. (B) At P7 electron micrographs from the contact region verify direct cellular appositions between the mossy fibers and the Purkinje cells. These contacts occur *en passant*, are densely packed with synaptic vesicles, and exhibit ultrastructural characteristics of synapses: synaptic vesicles polarized towards PSD-like structures on the Purkinje cell (blue arrowheads). (C) At P14 mossy fiber segments typically do not invade more than 15 µm into PCL. By electron microscopy the diameter of mossy fibers at the sites of contact with Purkinje cell soma is smaller compared to P7. Purkinje cell somata become surrounded by thin glial process, identified by very light cytoplasm and presence of glycogen particles. (D) At P21, none of the potential mossy fiber–Purkinje cell contacts identified by LM could be verified by EM as direct contacts, even though in some cases mossy fiber terminals were positioned as close as 150 nm from Purkinje cell soma. (E) Quantitation of correlated light-EM analysis. More than 90% of putative mossy fiber–Purkinje cell contacts identified by LM are direct cellular appositions as verified by serial EM at P0 and P7. At P14, only 40% of putative contacts are direct cellular appositions, and at P21 mossy fiber–Purkinje cell contacts are completely removed. Total of 55 potential contacts analyzed by serial EM analysis (P0, *n* = 13; P7, *n* = 21; P14, *n* = 13; P21, *n* = 8; taken from 34 areas, from 12 animals). (F) The filling fraction was calculated for somatic mossy fiber–Purkinje cell contacts by dividing the number of synapses by the number of direct contacts identified in the EM analysis. Synapses were defined by the presence of clustered synaptic vesicles docked at the plasma membrane opposite a postsynaptic density in the Purkinje cell. Since no direct mossy fiber–Purkinje cell contacts are detected at P21 no filling fraction can be calculated for this developmental time point.

Fifty-five putative contacts identified at the light microscopy level in P0, P7, P14, and P21 tissues were examined by electron microscopy. Tissue sections (50 µm) were re-sectioned into 7 µm semithin sections and re-examined again by light microscopy. Sections encompassing the putative mossy fiber–Purkinje cell contacts were then thin-sectioned (70 nm) and processed for ultrastructural analysis (Figure S2E–H). Cytological characteristics defined in previous studies allowed unambiguous identification of Purkinje and granule cell somata in electron micrographs, as well as other relevant cellular components of the cerebellar cortex (Figure S3) [3],[39]. Over 90% of putative contacts between mossy fibers and Purkinje cell somata identified by light microscopy at P0 and P7 indeed represent direct cellular appositions (Figure 3A,B,E). At P0, none of the mossy fiber–Purkinje cell (or mossy fiber–granule cell) contacts in the developing IGL/PCL had synaptic features, representing a filling fraction (synapses per contacts [6]) of 0.0 (Figure 3A,F). However, at P7 a substantial number of mossy fiber–Purkinje cell contacts exhibited ultrastructural characteristics of synapses (Figure 3B,E,F; several consecutive sections shown in Figure S4A, filling fraction = 0.37). At P14, direct contacts were still observed (5 direct contacts verified by EM of 13 putative contacts analyzed) but only one of them was synaptic (Figure 3C,F, filling fraction = 0.2). Finally, at P21 no direct mossy fiber–Purkinje cell contacts or synapses could be identified (Figure 3D).

During the apparent removal of contacts and the elimination of synapses between P7 and P14 we frequently observed mossy fibers separated from the Purkinje cell soma and/or ensheathed by glial processes (Figure S4C–F), reminiscent of pruning processes with axosome shedding observed in peripheral axons [40]–[42]. In addition, we observed instances where mossy fiber axons were engulfed by Purkinje cells (Figure S4C,D). Glial process ensheathing of Purkinje cells became even more prominent at P21. Some mossy fibers were positioned as close as 150 nm from the Purkinje cell soma (Figure 3D) but glial processes separated mossy fiber endings and the Purkinje cell soma. In summary, during the first 10 postnatal days approximately 30% of all labeled IGL mossy fibers derived from the PGN establish direct contacts with Purkinje cells. The quantitative analysis uncovers remarkable developmental changes in the “filling fraction”, i.e. the differentiation of direct mossy fiber–Purkinje cell contacts into synapses, rising from 0.0 at birth to 0.37 at postnatal day 7 (Figure 3F). In the second to third postnatal week, these synapses are eliminated and the contacts withdrawn, resulting in the selective innervation of granule and Golgi cells in the IGL. Notably, not all mossy fiber axons contact Purkinje cells. Therefore, specific signaling mechanisms must exist, first, to limit the invasion of pontine mossy fiber axons into the Purkinje cell territory during the first postnatal days and, second, to promote the removal of pontine mossy fiber–Purkinje cell synapses in the second postnatal week of development.

### Identification of Bone Morphogenetic Proteins as Candidate Retrograde Signals for Pontine Mossy Fiber Afferents

**Figure 4:**
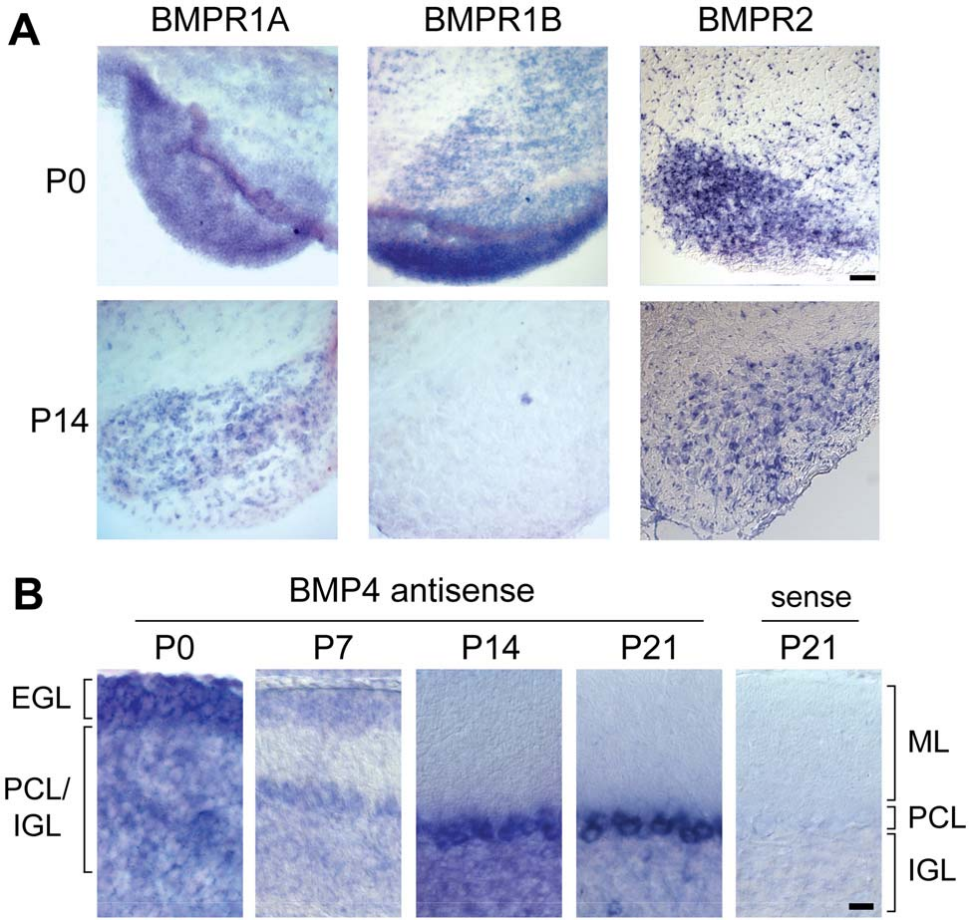
Expression of BMP signaling molecules in the pontocerebellar system. (A) In situ hybridization in the brainstem with BMPR1A, BMPR1B, and BMPR2 specific antisense probes. Images show an enlargement of sections through the pontine nucleus at P0 and P14. Scale bar: 200 µm. (B) In situ hybridization on cerebellar tissue (mediolateral cerebellum including SIM, Crus1, Crus2) at P0, P7, P14, P21 with a BMP4 specific antisense probe and sense control. Position of the external germinal layer (EGL), molecular layer (ML), Purkinje cell layer (PCL), and internal granular layer (IGL) are marked. Scale bar: 20 µm.

In *Drosophila melanogaster*, growth factors of the bone morphogenetic protein (BMP) family regulate synaptic growth, axon arborization, and synaptic homeostasis [27],[28],[43]–[45]. To explore whether a comparable signaling function might be conserved in the mouse cerebellum, we surveyed the expression of BMP signaling molecules in the developing ponto-cerebellar projection system. Using *in situ* hybridization, we detected mRNAs for BMP receptor 1A (BMPR1A), BMP receptor 1B (BMPR1B), and BMP receptor type 2 (BMPR2) in the PGN at P0, the time when pontine mossy fiber axons extend into the cerebellar cortex. By P14, detection of BMPR1B mRNA was reduced, while signals for BMPR1A and BMPR2 expression persist (Figure 4A). Within the cerebellar cortex significant expression of several BMP ligands was observed consistent with previous reports ([46]–[48] and unpublished data). We focused our analysis on BMP4 as it is highly expressed in Purkinje cells and dynamically regulated during the refinement of mossy fiber connectivity (Figure 4B). At P0, BMP4 mRNA is abundant in proliferating and premigratory granule cells of the EGL, and in scattered Purkinje cells (identified by their large diameter). BMP4 expression in Purkinje cells was reduced at P7, the time when mossy fiber–Purkinje cell synapses are most common, and expression was strongly up-regulated in Purkinje cells by postnatal day 14 (Figure 4B). At P21, BMP4 was highly expressed in Purkinje cells. In addition a subset of large diameter cells in the IGL (presumably Golgi cells) expressed BMP4. In summary, BMP4 and its signaling receptors are appropriately positioned to regulate mossy fiber target selection during postnatal development.

### Activation of the BMP Signaling Pathway in Pontine Neurons In Vivo

BMP-receptor activation results in phosphorylation of cytoplasmic SMAD proteins that translocate to the cell nucleus and activate transcription [49],[50]. Classical morphogenetic functions of BMPs depend on SMAD phosphorylation but phospho-SMAD (pSMAD)-independent BMP signaling read-outs have also been described [34],[51]–[53]. Robust SMAD phosphorylation was detected when recombinant BMP4 was added to cultured pontine explants in vitro and phosphorylation was prevented by co-application of the antagonist noggin (Figure S5A). Quantitative evaluation of SMAD phosphorylation in PGN in vivo using Western blot and immunohistochemistry revealed a dynamic regulation, with moderate levels at P0, strongly increased levels at P14, and persistent pSMAD immune-reactivity at P21 (Figure S5B–D). SMAD1,5,8 protein levels were not significantly altered during this developmental time period, suggesting that regulation of SMAD signaling occurs primarily at the level of SMAD phosphorylation (unpublished data). This demonstrates a functional BMP signaling pathway in developing pontine neurons in vitro and in vivo.

Given that BMP4 was dynamically expressed in Purkinje cells we asked whether Purkinje cell-derived BMP4 was required for SMAD activation in pontine neurons. We analyzed conditional *BMP4^fl/fl^::Pcp2^cre/cre^* knockout (BMP4 cKO) mice lacking BMP4 expression selectively in Purkinje cells. In the *Pcp2^cre^* knock-in line, cre-mediated recombination is detected during late embryonic stages and specifically in Purkinje cells ([54] and Figure S6). Ablation of BMP4 expression was verified by in situ hybridization (Figure S6C). Interestingly, SMAD activation in the pontine gray nucleus was not dramatically altered in BMP4 cKO mice (Figure S5D,E). While we cannot completely exclude that some of the pSMAD signal is due to incomplete ablation of the BMP4 expression in Purkinje cells, these results indicate that Purkinje cell-derived BMP4 might not be essential for pSMAD activation in pontine nuclei during postnatal development. Notably, Purkinje cells express significant amounts of BMP7 and other BMP growth factors during postnatal development which might be responsible for the persistent SMAD phosphorylation in the absence of BMP4 (unpublished data).

Importantly, signaling activities have been described for specific BMP growth factors that control cytoskeletal rearrangements through pSMAD-independent pathways [34],[51]–[53]. In commissural spinal neurons such BMP signals represent extrinsic cues for the initial polarization of axons [55],[56]. Therefore, we examined the possibility that target-derived BMP signaling might regulate axon development and axon-target interactions of pontine mossy fibers.

### BMP4 Is a Negative Regulator of Mossy Fiber Growth

**Figure 5:**
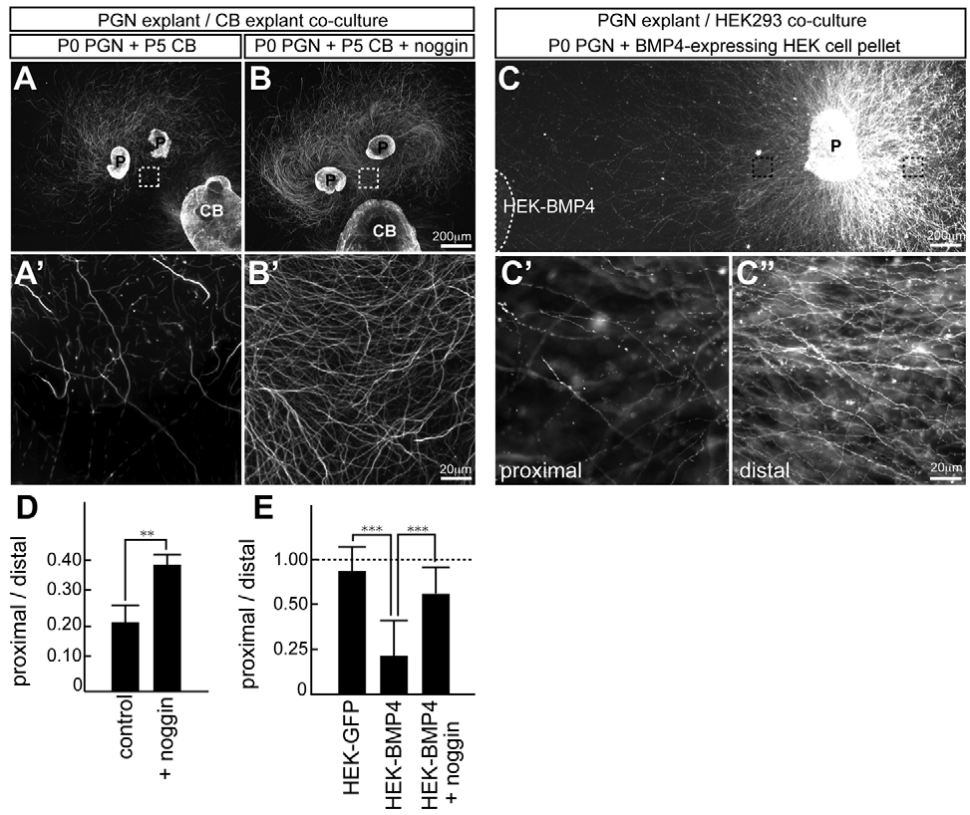
Cerebellum-derived BMP signal negatively regulates growth of mossy fiber axons in vitro. (A,B) Co-culture assay with pontine (P) and cerebellar (CB) explants. Axons are visualized with antibodies to the neuronal anti β3 tubulin subunit (Tuj1 antibody). Mossy fiber axon growth from PGN explants (P0+2 days in vitro) is reduced on the side facing cerebellar explant (panel A). Noggin (100 ng/ml) addition to the culture medium blocks the reduction in mossy fiber growth on the side facing cerebellar explant (B). Panels (A′) and (B′) show enlargements of the boxed areas in (A) and (B), respectively. (C) Collagen gel co-cultures of pontine explants (P) with BMP4-expressing HEK293 cell aggregates (BMP4-HEK) grown for 48 h. Panels in (C′) and (C″) are enlarged views of the boxed areas on the proximal and distal side with respect to the explant, respectively. (D) Quantification of axon density measured as Tuj1-positive area within fields 200 µm away from the explant. The measurements are shown as ratio of distal to proximal staining density. Growth inhibitory activity of cerebellar explants was counteracted by addition of 100 ng/ml noggin to the culture media (*n* = 29 explants from 3 experiments). (E) Quantification of axon density measured as Tuj1-positive area within fields 200 µm away from the explant. The measurements are shown as ratio of distal to proximal staining density. BMP4 axon growth inhibitory activity could be neutralized by addition of 100 ng/ml noggin to the culture media (*n* = 69 explants from 3 experiments). Statistical significance was determined by Mann-Whitney test. Two-tailed *p* values were used with 95% confidence intervals, ** *p*<0.01, *** *p*<0.001. Scale bars in (B) and (C): 200 µm; (B′) and (C″): 20 µm.

Previous work demonstrated that cerebellar explants cultured in vitro release a growth inhibiting activity for mossy fibers which is thought to resemble a target-derived stop signal for afferents [57]. Explants from the PGN exhibit robust radial axon outgrowth. However, when pontine explants are co-cultured with cerebellar tissue, axon growth on the side facing the cerebellar tissue is reduced, suggesting the presence of a growth inhibiting activity derived from cerebellar tissue (Figure 5A). In order to assess a possible role for BMPs in this process, we applied the soluble BMP antagonist noggin to the culture medium. Noggin addition blocked cerebellar growth retardation activity in this assay (Figure 5B,D). To directly examine whether BMP4 might exert such growth-inhibiting activity, we combined pontine explants with BMP4-expressing HEK293 cells in collagen gel co-cultures. Using this assay, we observed that BMP4 was sufficient to negatively regulate mossy fiber growth in vitro (Figure 5C). Importantly, this activity could be neutralized by addition of noggin to the collagen gel matrix, indicating that the growth regulation was indeed mediated by BMP signaling (Figure 5E). Therefore, BMP4 negatively regulates pontine mossy fiber growth in vitro.

### Purkinje Cell-Derived BMP4 Is a Negative Signal for Mossy Fiber Afferent-Target Interactions

Based on the dynamic regulation of BMP4 expression in Purkinje cells and the repulsive activity of BMP4 towards mossy fiber axons in vitro, we hypothesized that BMP4 might control either initial mossy fiber–Purkinje cell interactions, the detachment of mossy fiber–Purkinje cell contacts, or both. To explore these possibilities, we further examined the BMP4 cKO mice. Given the important patterning functions of BMP signaling in early cerebellar development [46],[47],[58] we first asked whether the overall anatomical organization of the cerebellar cortex or specification of cerebellar cell types was perturbed in the mutant mice. No significant changes were detected in the foliation pattern, cerebellar layering, specification of the major cell types, and expression of transcriptional markers and signaling molecules (Figure S7). In DiI labeled preparations, the number of labeled pontine mossy fiber axons or density of Purkinje cells observed in the cerebellar cortex of BMP4 cKO mice was not significantly different from control littermates or wild-type animals (Figure S7G and unpublished data). Finally, the development of climbing fibers and formation of vGlut2-positive climbing fiber synapses on the Purkinje cell dendrites was not noticeably altered in the BMP4 cKO mice (Figure S8).

**Figure 6:**
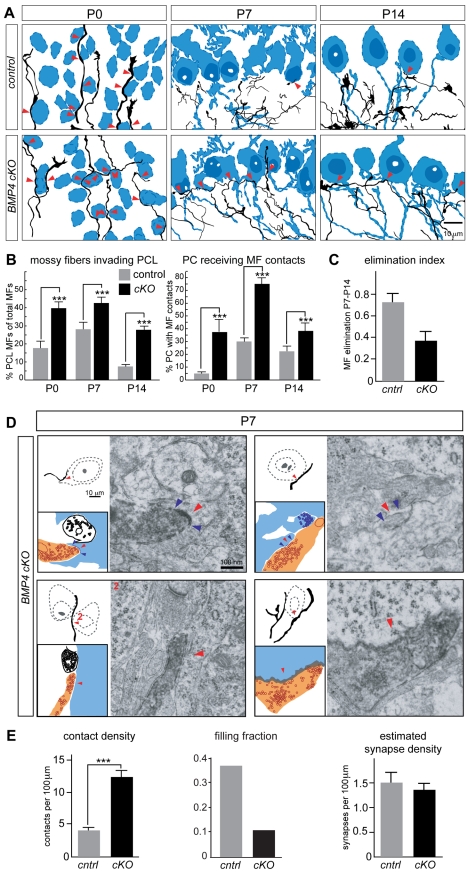
Excessive mossy fiber–Purkinje cell contacts and synapses in the absence of Purkinje cell-derived BMP4. (A) C*amera lucida* drawings of the upper IGL and the PCL of DiI-Calbindin double-labeled coronal sections from P0, P7, and P14 cerebellar hemispheres of littermate controls (*BMP4^fl/fl^*, upper panels) and BMP4 cKO mice (*BMP4^fl/fl^::Pcp2^cre/cre^* and *BMP4^fl/fl^::Pcp2^cre/+^*, lower panel). Purkinje cells are shown in blue. Red arrows point at putative contacts between mossy fibers and Purkinje cell soma. More mossy fiber segments extensively invade into the Purkinje cell territory in the BMP4 cKO tissue. (B) Quantitative analysis from *camera lucida* drawings of DiI-Calbindin double-labeled material showing significant increases in the percentage of mossy fiber segments entering the Purkinje cell territory and in the number of Purkinje cells receiving mossy fiber contacts (*n*>5,000 mossy fiber segments from 220 areas of 175 µm×120 µm×30 µm from 22 animals). Gray bars are control, black bars are cKO. ** *p*<0.01, *** *p*<0.001. (C) Elimination index for removal of mossy fibers from the PCL between P7 and P14 in control (gray) and *BMP4 cKO* mice (black bar). (D) Combinations of *camera lucida* drawings, schematic representations, and electron micrographs as in Figure 3. Two upper panels: At P7, many synaptic contacts in BMP cKO mice exhibit the same ultra-structural features as in the wild type. Lower left panel: Non-synaptic contacts between one mossy fiber segment and two neighboring Purkinje cells. Lower right panel: Some mutant contacts exhibit unusual ultra-structure with aggregated synaptic vesicles and ruffled plasma membranes at the contact site, characteristics never observed in wild-type or control mice. (E) Quantification of the density of EM-verified mossy fiber–Purkinje cell somatic contacts per 100 µm mossy fiber length in the PCL at P7 for BMP4^fl/fl^ control mice (gray) and BMP4cKO mice quantified by light microscopy (black). The filling fraction was substantially reduced in the BMP4 cKO tissue. Based on the contact density and filling fraction, an estimated synapse density was calculated which shows no significant difference in control and cKO mice. Data were collected from >3 animals per genotype, >50 areas per animal, >12 mossy fiber segments per area. *** *p*<0.001.

Next, we examined whether loss of Purkinje cell-derived BMP4 resulted in defects in mossy fiber–Purkinje cell contact formation, synapse formation, and/or synapse elimination. In BMP4 cKO mice the fraction of pontine mossy fibers that penetrated into the Purkinje cell territory at postnatal day 0 was increased approximately 2-fold as compared to control (Figure 6A,B). Moreover, the number of Purkinje cells receiving mossy fiber contacts was increased 7-fold at P0 and remained significantly increased over the following 2 wk. In our wild-type analysis (Figure 2) we identified a peak in mossy fiber elimination from the Purkinje cell territory in the second postnatal week. Mossy fiber elimination was quantitatively compared using an elimination index for the fraction of mossy fibers removed from the PCL between P7 and P14 ([MFs^PCL^ P7–MFs^PCL^ P14] / MFs^PCL^ P7). In the cKO animals elimination of mossy fibers still occurred but the elimination index for control and cKO mice was reduced to about 50% of that in control animals (Figure 6C). When normalized to the length of mossy fiber segments in the Purkinje cell territory, the density of contacts per 100 µm mossy fiber length was more than 3-fold increased at postnatal day 7 (Figure 6C). These observations highlight an essential function for BMP4 in the control of initial mossy fiber–Purkinje cell contact formation during the first postnatal week as well as the subsequent removal of mossy fiber processes from the Purkinje cell territory.

Considering the developmental regulation of the filling fraction observed in wild-type animals (Figure 3) we further examined mossy fiber–Purkinje cell contacts, synapses, and filling fractions at P7 using correlated light-electron microscopy. As in wild-type and control tissue, the majority of putative mossy fiber–Purkinje cell somatic contacts identified in BMP cKO mice indeed represented direct cellular appositions (38 direct contacts out of 40 potential contacts analyzed, Figure 6D,E). Some mutant contacts were characterized by unusual, irregular synapse-like profiles (Figures 6D, S4B). However, the filling fraction was substantially reduced in the cKO as only 11% of these contacts exhibited synaptic ultrastructure (Figure 6E). Based on the correlated light-EM analysis and the calculated filling fraction, the total density of mossy fiber–Purkinje cell synapses was not significantly changed, indicating that the excess contacts do not efficiently differentiate into synaptic structures. These experiments identify BMP4 as a retrograde signal that specifically controls mossy fiber-Purkinje cell contact formation and highlight that independent programs regulate contact versus synapse formation during postnatal development.

**Figure 7:**
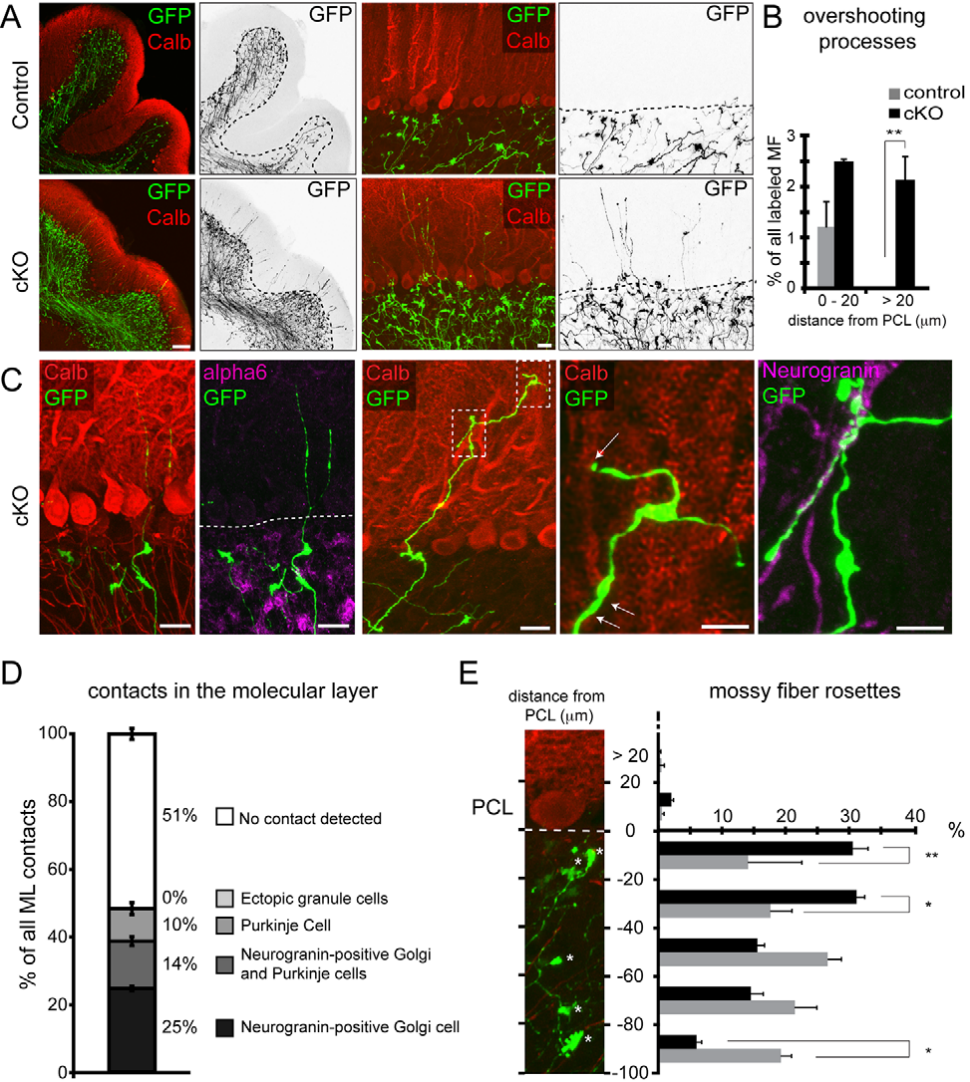
Mossy fiber defects in the mature cerebellum of BMP4 cKO mice. Pontine neurons in BMP4 cKO (*BMP4^fl/fl^::Pcp2^cre/cre^*) mice or control mice (*BMP4^+/+^::Pcp2^cre/cre^* and *BMP4^fl/+^::Pcp2^cre/+^*) were marked by *in utero* electroporation at E14.5 and analyzed at postnatal day 21. (A) Low magnification views of coronal sections show restriction of GFP-labeled mossy fibers to the IGL and overshooting of fibers in the BMP4 cKO mice. The inverted images show the GFP-filled mossy fiber axons in black, and the bottom of the Purkinje cell layer is marked with a dashed line. (B) Quantification of overshooting mossy fiber axons in the molecular layer within 0–20 µm or >20 µm distance from the bottom of the PCL in control (gray bars) and cKO mice (black bars). Fibers overshooting more than 20 µm beyond the PCL are never observed in control cerebella (surveyed in 60 sections through Crus1, Crus2, and simplex lobules from 3 electroporated control animals in this experimental series and sections from >20 control animals in other experiments). ** *p*<0.01. (C) Cellular contacts of overshooting mossy fibers (green) in tissue double labeled for the Purkinje cell marker calbindin (red), the mature granule cell marker GABA α6 (magenta), or the Golgi cell marker neurogranin (magenta). The right panels show higher magnification views of Purkinje and Golgi cell contacts. Some Purkinje cell contacts identified by three-dimensional evaluation of high-resolution confocal image stacks are marked with white arrows. (D) Frequency of contacts of molecular layer mossy fiber axons in BMP4 cKO cerebella was quantified for contacts with neurogranin-positive Golgi cells, calbindin-positive Purkinje cells, and GABA alpha6-positive granule cells. Single fibers that contact Golgi as well as Purkinje cells are listed as a separate group. Note that there are neurogranin-negative Golgi cells, which might be associated with mossy fibers leading to a possible underestimation of molecular layer cell contacts (*n* = 93 fibers from tissue obtained from three different BMP4 cKO animals). (E) The position of mossy fiber rosettes in GFP-positive pontine axons with respect to the bottom of the Purkinje cell layer was quantified. The percentage of fibers for each 20 µm segment away from the PCL is plotted (*n* = 393 fibers for control and *n* = 638 fibers for BMP4 cKO from 3 animals per genotype). Gray bars are control, and black bars are BMP4 cKO. Scale bars: (A) left panels 100 µm, right panels 20 µm; (C) left three panels 20 µm, two right panels 10 µm. * *p*<0.05, ** *p*<0.01.

If loss of BMP4 from Purkinje cells results in exuberant pontine mossy fiber–Purkinje cell contacts during early postnatal development, do these aberrant interactions perturb the placement or specificity of synapses in the mature cerebellum? Using *in utero* electroporation, we marked pontine mossy fibers in control and BMP4 cKO animals and examined their projection pattern at P21 when cerebellar development is essentially complete (Figure 7). While in wild-type mice mossy fibers were restricted to the IGL and did not protrude into the molecular layer, we observed overshooting axons in the BMP4 cKO mice (Figure 7A). A subset of mossy fiber axons penetrated more than 20 µm beyond the Purkinje cell somata into the molecular layer, a phenotype never observed in control cerebella (Figure 7B). Within the molecular layer, most mossy fiber axons had a smooth appearance but some developed swellings comparable to simple mossy fiber rosettes. Overshooting mossy fiber axons have been observed previously in mouse mutants with perturbed granule cell migration [59]. However, we did not observe ectopic granule cells in the molecular layer of BMP4 cKO mice (Figure 7C). Instead, high-resolution analysis of the overshooting mossy fiber axons revealed that some established direct contacts with the dendritic tree of Purkinje cells. Other overshooting axons formed contacts with neurogranin-positive Golgi cells (Figure 7C). Notably, Golgi cells are one of the specific synaptic targets of ponto-cerebellar mossy fibers in the IGL. However, in wild-type mice mossy fiber synapses are excluded from the distal dendritic arbors in the IGL. Finally, we examined the position of pontine mossy fiber synapses in the IGL and observed a significant shift of mossy fiber rosettes towards the PCL (within 40 µm of the Purkinje cell somata, Figure 7). Therefore, loss of Purkinje cell-derived BMP4 results in persistent alterations in mossy fiber connectivity in the mature cerebellum.

## Discussion

Cerebellar circuits and the “crystalline” architecture of the cerebellar cortex are a prime example of the precision of neuronal connectivity. In this study, we identified cellular and molecular mechanisms orchestrating aspects of afferent-target specificity in cerebellar networks. First, we demonstrate that synaptic specificity of pontine mossy fibers emerges in a protracted, stepwise process that encompasses extensive contacts and synapse formation with Purkinje cells. Second, we identify BMP4 as a retrograde, Purkinje cell-derived signal that negatively regulates mossy fiber-Purkinje cell contacts and synaptic specificity.

### BMP Growth Factors as Retrograde Signals

BMPs are key regulators of patterning and cell fate decisions, but novel functions in neuronal wiring are emerging [49],[60]–[62]. In the vertebrate central nervous system BMPs (and the related TGFbeta growth factors) control initial axon orientation and axon regeneration [55],[56],[63]–[65]. Moreover, retrograde, target-derived BMP signaling has been examined in the peripheral nervous system [66]–[69]. At the *Drosophila* neuromuscular junction a muscle-derived BMP-analogue regulates synaptic growth and homeostatic signaling [27],[29],[30],[43],[70]. Whether BMP growth factors have similar retrograde signaling activities in the central nervous system and, specifically in axon-target interactions in vertebrates, has remained unclear. In our experiments, we explored retrograde BMP signaling in the mouse pontocerebellar system and uncovered a novel function during the development of synaptic target specificity. In this system, BMP4 acts as a negative signal that limits interactions of mossy fibers with Purkinje cells, a transient target cell. The dynamic regulation of BMP4 expression in Purkinje cells mirrors the assembly of mossy fiber-Purkinje cell contacts and synapses, with a transient peak at P7 where BMP4 expression is low. Thereafter, BMP4 is strongly up-regulated and mossy fiber-Purkinje cell contacts are eliminated.

### Regulation of Mossy Fiber Target Specificity by BMP4

The mossy fiber phenotypes in the BMP4 cKO mice highlight a critical function of Purkinje cell-derived BMP4 in mossy fiber–Purkinje cell interactions. In the cKO mice, there is a substantial increase in mossy fiber–Purkinje cell contacts at early postnatal stages (P0–P7). This supports an essential repulsive role for BMP4 in target recognition which limits the initial mossy fiber–Purkinje cell contacts and restricts the invading mossy fiber axons to their target territory in the IGL. The correlated light-electron microscopy analysis enabled us to dissociate changes in contact and synapse formation in the cerebellar system. Notably, while BMP4 cKO mice exhibit a 3-fold increase in mossy fiber–Purkinje cell contact density we did not detect a comparable increase in synapse density. Therefore, axon target contacts and synapse formation are controlled by different signaling systems.

The subsequent, removal of mossy fiber–Purkinje cell contacts and elimination of mossy fiber processes from the Purkinje cell territory was significantly delayed, and after completion of cerebellar development, we observed persistent overshooting mossy fiber projections in the Purkinje cell and molecular layers. Some overshooting axons retain interactions with Purkinje cells, while others form contacts on distal Golgi cell dendrites. Notably, Golgi cells are appropriate synaptic partners of mossy fibers, but in BMP4 cKO cerebella mossy fiber–Golgi cell interactions are observed ectopically in the molecular layer. These findings support an important role for Purkinje cell-derived BMP4 in eliminating mossy fiber projections from this area, in addition to its function in regulation of the early mossy fiber–Purkinje cell contacts.

Within the IGL, the placement of mossy fiber rosettes was shifted towards the Purkinje cell layer, further supporting a repulsive role for Purkinje cell-derived BMP4. However, the fact that most mossy fiber axons did not overshoot to the molecular layer indicates that there are additional signals that restrict mossy fibers to the IGL. BMP2 and 7 transcripts are up-regulated in Purkinje cells of the cKO mice (unpublished data) and may partially compensate for the loss of BMP4. Moreover, the specificity of mossy fiber connectivity is likely to emerge not only from negative, Purkinje cell-derived signals but from an interplay with positive signals derived from the appropriate target cells. Granule cells express FGF22, Wnt7a, and neuroligins which all have been demonstrated to have positive, synaptogenic activities towards mossy fiber afferents [16]–[18]. Therefore, presentation of these synaptogenic signals by mature granule cells which strongly increase in number at later postnatal stages (P7–P21) may compete with the constant number of Purkinje cells for mossy fiber contact. A prediction of this model is that direct mossy fiber-Purkinje cell synapses would persist in the absence of granule cells. This is, indeed, observed in agranular cerebella of mouse mutants or after irradiation where mossy fiber target selectivity can be examined in the absence of the appropriate synaptic targets [71]. Importantly, while most mossy fibers were appropriately restricted to the IGL, the positioning of mossy fiber rosettes within the IGL was shifted closer to the Purkinje cell layer (Figure 7D), consistent with the loss of a negative regulator of synaptic connectivity in Purkinje cells of BMP4 cKO mice.

### Transient Target Cells in the Development of Neuronal Connectivity

The finding that the development of mossy fiber target specificity involves not only extensive contact but also synapse formation with Purkinje cells argues against a model of absolute recognition specificity for unique synaptic targets. This remodeling of transient target interactions is reminiscent of interactions in the thalamo-cortical projection and for *Cajal Retzius* cells in the hippocampus [72]–[74]. In both cases, afferents enter the target territory before their appropriate target cells have fully differentiated and form transient synapses on a third cell type (subplate neurons and *Cajal Retzius* cells, respectively). This situation in the hippocampus is comparable to the transient mossy fiber–Purkinje cell synapses described in our study that are elaborated during early postnatal development when only few granule cells have descended into the forming IGL. While the initial assembly of such transient contacts is comparable, the mechanism of contact removal is fundamentally different. Elimination of transient synapses received by subplate and *Cajal Retzius* neurons occurs via programmed cell death of the transient target cells. By contrast, removal of mossy fiber–Purkinje cell interactions occurs independently of Purkinje cell death and requires signals for contact destabilization.

### Bug or Feature?

The existence of widespread mossy fiber–Purkinje cell interactions during development poses the question of whether these synapses simply represent an imprecision in the initial trans-synaptic interactions or whether transient contacts serve a purpose in the development of functional cerebellar circuits. In the hippocampus, *Cajal Retzius* cells appear to be required for the laminar specificity of entorhinal axon projections [74]. Similarly, in the absence of subplate neurons, thalamocortical axons do not establish appropriate synaptic connectivity [75],[76]. Therefore, transient mossy fiber–Purkinje cell interactions might similarly contribute to the assembly of cerebellar circuits. The cerebellar cortex is subdivided into longitudinal bands identified by specific molecular codes of gene expression in Purkinje cells [77],[78]. This code develops during the first postnatal weeks, coincident with emergence of mature cellular and sub-cellular targeting specificity of both climbing and mossy fiber afferents. Recent tracing studies indicate that there is a precise somatotopic matching of pontine and climbing fibers [79],[80]. This raises the possibility that transient mossy fiber–Purkinje cell interactions might provide a mechanism to coordinate mossy fiber and climbing fiber development and, thereby, serve a functional role in the assembly of cerebellar circuits.

## Materials and Methods

### Mouse Strains

All animal experiments were reviewed and approved by the institutional animal care and use committee of Columbia University and the cantonal veterinary office Basel, respectively. Mice were of the NMRI (Figure 1) and C57BL/6 strains (all other experiments). PCP2^cre^ knock-in mice were previously described [54]. The conditional BMP4 floxed allele (BMP4^fl^) was generously provided by Dr. Brigid Hogan [81]. Htr5b-GFP mice are BAC transgenic mice generated by the GENSAT consortium [82] and were obtained from the MMRRC repository. Thy1.2-GFP (GFP-O) mice were generated by Drs. Sanes and Feng [38] and were obtained from the Jackson Laboratory. R26-lox-stop-lox-YFP were described in [83].

### In Utero Electroporation of Precerebellar Neuron Precursors

Timed-pregnant mice (NMRI or C57BL6 background) were used at embryonic day 14.5 following the protocol described in [37]. After electroporation, the mice were brought to term, pups were sacrificed by transcardial perfusion with 4% paraformaldehyde in 100 mM Na-phosphate buffer (pH7.4), and tissue from successfully electroporated pups (P7, P14, P21) was processed for immunohistochemistry.

### Western Blotting, Immunohistochemistry, In Situ Hybridization, and In Vitro Assays

The following primary antibodies were used in this study: rabbit anti-Shank1a [84], goat anti-Car8 (Frontiers Institute), rabbit and mouse anti-Calbindin D-28K (Swant), rabbit anti-GFP [85], rabbit anti Pax6 (Covance), guinea pig anti-vGlut1 (Chemicon), mouse anti-vGlut2 (Chemicon), goat anti-Parvalbumin (Swant), rabbit anti-Smad1 (Millipore), rabbit anti-phospho-SMAD1/5/8 (Millipore), rabbit anti-SMAD1,5,8 (Imgenex), mouse anti-NeuN (Millipore), rabbit anti-neurogranin (Abcam), mouse anti-actin (Sigma), and mouse anti-VAMP2 (Synaptic Systems).

Most procedures followed standard protocols; see Text S1 for details.

### Confocal Microscopy Analysis

High-resolution images of 30 to 40 µm z-stacks consisting of 0.45 µm thick optical sections were acquired using Zeiss LSM510, a Zeiss LSM5 Exciter, or a LIS-spinning disk confocal system. Direct apposition of cellular markers was identified by rotating the 3D reconstruction of the stacks using Imaris Software (Bitplane).

Quantitative assessment of SMAD1,5,8 activation was performed using pSMAD1,5,8 and NeuN immunolabelling on 50 µm thick sagittal section using Metamorph software. The percentage of pSMAD1,5,8 positive cells among the NeuN positive cells and the pSMAD1,5,8 fluorescence intensity per NeuN area was determined through intensity thresholding and integrated morphometry analysis using MetaMorph software.

### Correlated Light–Electron Microscopy Analysis

DiI (1,1′-dioctadecyl-3,3,3′,3′-tetraindocarboyanine perchlorate, Molecular Probes) labeling was modified from a previously published procedure [86]; see Text S1 for details.

Quantitative assessment of mossy fiber invasion into Purkinje cell territory was performed on *camera lucida* drawings of DiI and calbindin double-labeled material (50 µm coronal “thick” sections, 100× objective) of P0, P7, P14, and P21 cerebellar hemispheres (crus1, crus2, and simplex lobules). All *camera lucida* drawings contained all of the labeled mossy fiber segments and Purkinje cell outlines drawn from a fixed area size of 175 µm horizontal×120 µm vertical×30 µm deep (thickness of one Purkinje cell soma), encompassing the upper IGL and PCL, and spanning a stretch containing on average 40 Purkinje cells at P0 (before PC alignment occurs), and 9 Purkinje cells at P7–P21. For the sake of consistency, and since Purkinje cell density and the angle of the mossy fiber segments penetrating the PCL differs at the base, versus apex, versus sides of the folia, areas for analysis were always drawn from the sides of the folia. The percentage of mossy fiber segments invading into the PCL out of all mossy fiber segments drawn per area was scored (>20 segments per area from >25 areas obtained from 5–9 animals per time point, >3 50 µm section per animal were analyzed). For the quantification of Purkinje cells receiving putative somatic contacts from mossy fibers, contacts were judged as varicosities in the mossy fiber axon at the site apparently immediately adjacent to Purkinje cell soma. For quantification of contact density per mossy fiber length (in Figure 7D) *camera lucida* drawings were scanned at 600 dpi, and the length of mossy fiber segments in the PCL was measured using line tool in NeuronJ [87]. The number of putative contacts on Purkinje cell somata per mossy fiber segment was scored visually, using the criteria described above.

The filling fraction was calculated as actual synapses divided by the number of contacts (EM-verified). The elimination index for mossy fibers projecting into the Purkinje cell layer (MFs^PCL^) was calculated using the data points for P7 and P14 (graph Figure 6B) as follows: [MFs^PCL^ P7–MFs^PCL^ P14] / MFs^PCL^ P7.

## Supporting Information

Text S1 Supplemental material and methods. Western blotting, immunohistochemistry, and in situ hybridization; functional in vitro assays; correlated light–electron microscopy analysis; supplemental references.
(DOC)

Figure S1 Marking of mossy fiber afferents by *in utero* electroporation and in transgenic mice. (A) Dorsal view of an E14.5 mouse embryo and schematic drawing of the neural tube at this stage. The fourth ventricle, the site of DNA injection for the *in utero* electroporation, is marked. DNA is delivered unilaterally into precursor cells flanking one side of the ventricle. (B) Coronal sections of pontine gray nucleus and inferior olivary nucleus from electroporated mice (P7) demonstrating selective marking of pontine gray nucleus neurons and absence of labeling in the inferior olive. (C) Pontine mossy fiber axons marked by EGFP expression (green) introduced by *in utero* electroporation and analyzed at P21. Purkinje cells are marked with antibodies to calbindin (red). (D) Schematic diagram illustrating widespread GFP-positive brainstem areas (green shading) in GFP-O transgenic mice at postnatal day 14. Red dashed lines indicate planes of coronal tissue sections shown in (E) and (F). (E) GFP fluorescence in a coronal section through P14 brainstem is observed in few cell bodies located in the Pontine Gray Nucleus (PGN) and in a significant number of cell bodies of the Oral Pontine Reticular Nucleus (PnO) amongst other brainstem nuclei (not shown). (F) Coronal section through the cerebellar hemisphere (P21) showing calbindin-labeled Purkinje cells (red) and GFP-labeled fibers (green) with typical mossy fiber morphology in the WM and the IGL. Labeling in the cerebellar hemispheres of GFP-O mice is mostly restricted to mossy fibers. (G) Direct contacts between mossy fibers (green) and Purkinje cell soma (anti-calbindin, red) and axons (P14). The middle panel shows an enlargement of a contact. The right panel represents a projection of the confocal image stack rotated by 90° confirming the juxtaposition of mossy fiber ending and Purkinje cell soma. Scale bars: (B) upper panel 500 µm, lower panel 200 µm; (C) 20 µm; (F) 100 µm.
(TIF)

Figure S2 Correlated light and electron microscopic analysis. (A–D) Photographs and *camera lucida* tracings of the IGL and PCL in DiI labeled 50 µm thick sections from P0, P7, P14, and P21 time points. In the photographs a single plane of focus is seen, such that labeled mossy fibers appear as discontinuous segments (demarcated by red arrowheads). Somata of Purkinje and granule cells can be identified by DIC microscopy. The two cell types can be distinguished by unique cytological profiles (soma diameter, nucleoli, density of cytoplasm and nuclei). Corresponding *camera lucida* drawings, reconstructing mossy fiber arbors through the Z-planes of the 50 µm thick sections, are shown to the right side of the photographs (red arrowheads demarcate the same mossy fiber segments as in the photographs). Black asterisk indicates blood vessel. Scale bar: 10 µm. (E–H) Analysis steps in correlated light-electron microscopy. (E) Putative contacts (a1, a2, and b) between pontine mossy fibers and Purkinje cell somata identified from *camera lucida* tracings of 50 µm thick section. (F) Photograph of 7 µm semithin section of corresponding area verifying that putative contacts a1, a2, and b are indeed immediately adjacent to the soma of Purkinje cells A and B. (G) Electron micrographs of 70 nm thin sections corresponding to the same area as in (E) and (F). Cellular landmarks, such as blood vessels, or Purkinje and granule cell nuclei and nucleoli, allow correlation of cellular features and relationships identified at light level with the ultrastructural profiles. (H) Electron micrographs of contacts a1, a2, and b taken at 30,000× magnification showing that these are direct cellular appositions between mossy fibers and Purkinje cell somata (a1 and b) and on perisomatic protrusions (a2), respectively. All three contacts show ultrastructural features of synapses (polarization of synaptic vesicles and PSDs). (I,J,K) DiI-calbindin double-labeled sections. Calbindin immunohistochemistry facilitates identification of Purkinje cell domains into which mossy fibers project. Panel (K) provides an example of a *camera lucida* drawing of mossy fiber contacts with five Purkinje cells (a–e). Scale bars: (A, E) 10 µm; (G) 5 µm ; (H) 500nm.
(TIF)

Figure S3 The cytological features used for unambiguous identification of Purkinje cells and mossy fiber processes in the correlated light–EM analysis. (A) C*amera lucida* tracing showing mossy fiber segments (black) and outlines of Purkinje cell somata, nuclei, and nucleoli (blue dotted lines) identified by DIC microscopy. Drawings obtain with a *camera lucida* and 100× objective in DiI-labeled 50 µm thick section from P14 cerebellum. Area boxed in red contains a rosette (highlighted by red arrow), a filopodia-like protrusion (red arrowhead), and establishes a putative contact with the soma of a Purkinje cell. Scale bar: 10 µm. (B) Electron micrograph of a 70 nm thin section corresponding to the boxed area in (A). The labeled mossy fiber including the portion of the rosette is outlined by a dashed orange line, and a Purkinje cell soma is outlined in blue. Cerebellar cell types can be distinguished in electron micrographs by unique ultrastructural features: clusters of polyribosomes (blue arrow) in the Purkinje cell somatic cytoplasm; dark, densely packed chromatin (green N) distinguishes granule cell nuclei; very light cytoplasm and glycogen particles (yellow arrow) distinguish glial cell processes. Scale bar: 2 µm. (C) Electron micrograph of the IGL/PCL boundary containing seven unlabeled and one labeled (asterisk) mossy fiber rosettes highlighted by orange overlay. Mossy fiber rosettes are distinguished by their large diameter, irregular outline, and abundance of synaptic vesicles, mitochondria, and multiple active zones juxtaposed to PSDs on the surrounding processes. Scale bar: 2 µm. (D) Higher magnification of an unlabeled (left) and labeled (right, asterisk) mossy fiber rosette (orange overlay and red arrows). Even though all the lipid membranes appear darker due to incorporation of DiI, the ultrastructural characteristics are identical to unlabeled rosettes. Scale bar: 500 nm. (E) DiI-labeled thin mossy fiber processes in the upper IGL, near but not in contact with the Purkinje cell soma. These thin protrusions are filled with synaptic vesicles and establish multiple synaptic contacts with surrounding processes. Scale bar: 500 nm.
(TIF)

Figure S4 Ultrastructural features of mossy fiber–Purkinje cell interactions. (A) Serial sections of the mossy fiber–Purkinje cell synaptic contact in wild-type cerebellum shown in Figure 3B (P7). (B) Serial sections of the mossy fiber–Purkinje cell contact shown in Figure 7E in BMP4 cKO mouse cerebellum (P7). Note that pre- and postsynaptic densities are often lacking, the membrane between the mossy fiber and Purkinje cell soma is ruffled, and synaptic vesicles are irregularly shaped. (C–D) Electron micrograph (P14) showing engulfment of DiI-labeled mossy fiber process in Purkinje cell (blue overlay, red arrows, one revealing an endocytotic figure nearby). Thin glial processes can frequently be seen separating mossy fibers from the Purkinje cell soma (yellow arrow, C). (E–F) Glial ensheathment of mossy fiber processes near Purkinje cell soma in P14 wild-type cerebellum. Glial processes (identified by the presence of glycogen particles in the cytoplasm, yellow overlay) can frequently be seen to separate mossy processes from the Purkinje cell soma. Scale bar: 500 nm; “N” marks granule cell nuclei (green).
(TIF)

Figure S5 Developmental analysis of SMAD phosphorylation in pontine gray nuclei. (A) Activation of the BMP signaling pathway in pontine explants cultured in vitro. Western blots for the phosphorylated forms of SMAD1, 5, and 8 (pSMAD1,5,8) in PGN explants (2 d in vitro), treated with recombinant BMP4 (10 ng/ml) and/or noggin (100 ng/ml). (B) Western blots for SMAD1, 5, and 8 (SMAD1,5,8), their phosphorylated forms (pSMAD1,5,8), and actin in the PGN at P0, P3, P7, P10, P14, P17, and P21, showing dynamic changes in the levels of pSMAD1,5,8 in the PGN during development. The graph shows a quantitative assessment of SMAD1,5,8 activation during development measured by Western blot and expressed as a ratio of pSMAD1,5,8 to actin immune-reactivity in the cell extracts. The intensity ratio obtained for the P0 time point was set to “one” and measurements for other developmental time points are expressed relative to P0. (C) Immunohistochemistry for pSMAD1,5,8 (red) and NeuN (green) detected in parasagittal sections through the PGN at P0, P7, P14, and P21. Note the increase in number and intensity of pSMAD1,5,8 nuclear labeling at P14 and P21 compared to P7. Scale bar: 10 µm. (D) Comparison of SMAD1,5,8 activation in control (*BMP4^+/+^::Pcp2^cre/cre^*) and cKO (*BMP4^fl/fl^::Pcp2^cre/cre^*) at P7, P14, and P21 in the PGN assessed by immunohistochemistry. PGN sections were immunolabelled for pSMAD1,5,8 and NeuN (a marker of pontine projection neurons). Left graph shows the percentage of pSMAD1,5,8-positive cells amongst the NeuN-positive neurons and right graph shows the average intensity of the nuclear pSMAD1,5,8 labeling (poor NeuN immune-reactivity at P0 precluded quantitative analysis of this earliest time point). Gray bars are control, black bars are cKO. * *p*<0.05, ** *p*<0.01, *** *p*<0.001. (E) Western blot analysis of pSMAD1,5,8 levels in pontine nuclei at P14 and P21 reveals no significant differences in *BMP4^+/+^::Pcp2^cre/cre^* (control) and *BMP4^fl/fl^::Pcp2^cre/cre^* (BMP4 cKO) mice. pSMAD levels were compared to actin immunoreactivity in the same sample. pSMAD∶actin ratios were arbitrarily set to “one” for the P14 control sample in this experiment.
(TIF)

Figure S6 Conditional ablation of BMP4 using Pcp2cre KI mice. BMP4 conditional KO mice were obtained by crossing a floxed BMP4 allele [81] and Pcp2 cre knock-in mice [54] which express cre recombinase from the Pcp2 locus. (A) The cell-type specificity of cre-mediated recombination in Pcp2-cre knock-in mice was confirmed using a ROSA26-lox-stop-lox-YFP reporter [83]. Recombination is observed specifically in Purkinje cells (co-labeled with calbindin which labels a subset and RORalpha which labels all Purkinje cells) but not in the brain stem. Note that at P0 recombination in Purkinje cells throughout the cerebellum is detected in coronal sections. Higher magnification views (lower row) reveal that at least 50% of RORalpha positive Purkinje cells show activation of the reporter. (B) Schematic drawing of the conditional BMP4 locus. Exons 3 and 4 are flanked by loxP sites. (C) Confirmation of BMP4 expression in Purkinje cells (P14) by in situ hybridization with a probe for the ablated exon 3. Individual Purkinje cells are marked by arrowheads. Note that the high sequence similarity between BMP4 exon 3 and other BMP family members makes detection more challenging than in the experiments using probes against the UTR used in Figure 4. (D) Visualization of cre-mediated recombination in Purkinje cells at P14 using a ROSA26-lox-stop-lox-nuclear lacZ reporter allele. Purkinje cells are labeled with anti-calbindin antibodies. Scale bars: (C) 30 µm; (D) 20 µm.
(TIF)

Figure S7 Characterization of BMP4 cKO cerebellum. (A) Normal morphology and layering of the cerebellum in P21 wild-type and BMP4 cKO mice analyzed by Nissl staining. Representative sections of the mediolateral cerebellum are shown. Scale bar: 250 µm. (B) No significant alterations in the thickness of EGL, IGL, and WM in wild-type and BMP4 cKO mice. Thickness of layers was measured in Hoechst-stained parasagittal sections from P7 animals and relative thickness of external germinal layer (EGL), Purkinje cell layer (PCL), and internal granular layer (IGL) was measured (*n*=8 sections from 2 animals for each genotype). (C) Stellate and basket cell interneurons were identified as parvalbumin (red)-positive and calbindin (green)-negative cells. The density and morphology was not noticeably altered in cerebella of BMP4 cKO mice as compared to wild-type. (D) Cell densities of parvalbumin-positive/calbindin-negative cells (Parv^+^Calb^−^) were quantified from 6–7 fields per genotype from wild-type and BMP4 cKO animals. Cell numbers per area of molecular layer (in mm^2^) are compared. (E) The pontine projection neuron markers Bar-Homologue-like 1 (BarHl1), Pax6, and Zic1 are expressed in pontine nuclei from mutant mice. Images show in situ hybridization (BarHl1) and immunohistochemistry (anti-Pax6 and anti-Zic1) in BMP4 conditional mutant mice at P0 time point. Scale bar: 100 µm. (F) Expression of wnt3a and shh mRNAs in heterozygous (*BMP4^fl/+^::Pcp2^cre/+^*) and homozygous (*BMP4^fl/fl^::Pcp2^cre/+^*) conditional mutant cerebella (P14) detected by in situ hybridization. Sense control probes are shown on the left. Scale bar: 20 µm. (G) Quantitative analysis of mossy fiber and Purkinje cell numbers from *camera lucida* drawings (encompassing all of the mossy fiber segments and Purkinje cell outlines reconstructed from an area (175 µm×120 µm×30 µm), oriented parallel to the PCL, and recorded with a 100× objective) of P0, P7, and P14 time points from control littermates (BMP4fl/fl) and BMP4 cKO animals (>15 segments from >9 fields from >3 sections, >2 control, and >3 cKO animals per time point). There are no significant differences between genotypes.
(TIF)

Figure S8 Climbing fiber morphology is not significantly altered in BMP4 cKO mice. The BAC transgenic line Htr5b-GFP shows expression of GFP in a subset of inferior olivary neurons starting at P14 and was used to examine climbing fiber morphology. Sagittal sections of P7 (A, B), P14 (C, D), and P21 (E, F) of control (upper panels, *BMP4^+l+^::Pcp2^cre/cre^*) and BMP4 cKO (lower panels, *BMP4^fllfl^::Pcp2^cre/cre^*) cerebellar cortex. (A′, B′, C′, D′, E′, F′) show enlargements and demonstrate similar climbing fiber (anti-GFP, green in C, D, E and F) morphology, presynaptic synaptic vesicle accumulation (anti-vGlut2, magenta), and arborization along the Purkinje cell dendrites (anti-Car8, green in A, B, and red in C, D, E, F) in control and mutant cerebella. Scale bars: (B, D, F) 10 µm; (B′, D′, F′) 5 µm.
(TIF)

## Abbreviations

BMP: bone morphogenetic protein
BMPR: BMP receptor
IGL: inner granular layer
PGN: pontine gray nucleus
pSMAD: phospho-SMAD
