# Supplementary material for: Development of Axon-Target Specificity of Ponto-Cerebellar Afferents
Source: PLoS Biol. 2011 Feb 8;9(2):e1001013. doi: 10.1371/journal.pbio.1001013 (PMC3035609; doi:10.1371/journal.pbio.1001013)
Supplement: Text S1 — Supplemental material and methods. Western blotting, immunohistochemistry, and in situ hybridization; functional in vitro assays; correlated light–electron microscopy analysis; supplemental references. (DOC) [file pbio.1001013.s001.doc]

**Supplemental Materials and Methods**

***Western blotting and immunohistochemistry, in situ hybridization***

For western blotting analysis, pontine gray nuclei or pontine explants were harvested and homogenized in 10% (tissue weight/buffer volume) lysis buffer [100 mM NaCl, 20 mM Tris-HCl, pH 8.0, 1mM EDTA, 1% (w/v) Triton X-100, 0.2% (w/v) SDS, 2 mM dithiothreitol, 50mM sodium fluoride, 1mM beta-glycerophosphate, 1mM sodium orthovanadate, complete protease inhibitor cocktail (Roche)]; the protein concentration was measured with a BCA protein assay (Pierce). For the analysis of SMAD phosphorylation, twenty micrograms of total protein were loaded into each lane, transferred to nitrocellulose and detected using enhanced chemoluminescent detection (Pierce).

For immunohistochemistry, brains (P0) were fixed by immersion in 4% paraformaldehyde (PFA) in 0.12 M phosphate buffer, pH 7.4, or by transcardial perfusion with 4% PFA (mice older than P0), brains were removed and postfixed overnight. Brains were sectioned on vibratome or cryostat, blocked in 10% Donkey serum or BSA, depending on the antigens. For the Shank1a staining epitope retrieval with pepsin digestion, and for Pax6 staining epitope retrieval by microwaving in Citric acid solution were used. In all staining we used species-specific, highly cross-absorbed secondary antibodies conjugated to fluorophores Cy2, Cy3, or Cy5 (Jackson ImmunoResearch).

In situ hybridizations were performed on 50µm floating vibratome sections using digoxigenin-labeled mRNA probes following standard protocol (Schaeren-Wiemers and Gerfin-Moser, 1993). 200-400bp probes derived from the 5’UTR sequences of different BMPs were used to minimize cross-reactivity between individual BMP isoforms. A Barhl1 probe was kindly provided by Dr. Susan Dymecki, BMPR1a, BMPR1a, and BMPR2 probes were kindly provided by Dr. Edward Laufer.

***Functional in vitro assays***

## Co-culture of pontine and cerebellar explants was slightly modified from previous protocols (Baird et al., 1992; Zhang and Mason, 1998). Basilar pontine nuclei were dissected from P0 mice in DMEM media and were cut into 8-10 approximately pieces of 100µm^2^ diameter. Cerebellar explants were prepared from P5 mouse lateral cerebella. The lateral cerebellum was pinched out using #55 forceps, and was further cut into individual folia, which were sliced into approximately 300µm^2^ pieces using a wire tool. One cerebellar explant was plated in the center of a laminin-coated coverslip, surrounded by four pontine explants (distance of 300 - 600μm). Cultures were grown in 5% CO_2_ atmosphere at 37^°C^ for 48 hrs in Neurobasal media containing Penicillin-Streptomycin, 2mM Glutamate, and B27 supplement (Invitrogen).

## For the collagen gel culture assay of pontine explants and BMP-HEK293 cell pellets HEK293 cells at about 70% confluency were transfected with a BMP4-myc (kindly provided by Dr. Jane Dodd) or control EGFP expression vector using FuGENE reagent (Roche). On the following day the cells were gently trypsinized, collected by centrifugation, and resuspended at 2.5 X 10^6^ cells/ml. To balance the level of BMP4 released from the HEK293 cell aggregate, transfected cells were mixed with untransfected cells at a ratio of 1:60. As previously observed in other chemotrophic assays with BMP growth factors, higher BMP4 levels showed less axon growth retardation activities, presumably due to desensitization of the receptors. 20µl hanging drops of the cell-suspension were plated on the inner surface of 5cm culture dish lids with the dishes filled with 2ml of DMEM culture media. The drops were incubated inverted in 5% CO_2_ atmosphere at 37°C for 3hr. During the first 30 minutes a pen was used to tap on the lids every 5-10 minutes to facilitate aggregation of the cells into pellets. This protocol yielded firm round aggregates of HEK cells. Each aggregate was cut under a dissection microscope into 8 pieces of approximately 300µm^2^ with a wire tool. One HEK293 cell pellet piece was placed in the middle of a collagen cushion, surrounded by three pontine explants. To confirm bioactivity of the expressed BMP growth factors, dorsal cell fate induction was monitored in chick neural plate explants that were combined with the HEK293 cell aggregates. Each explant was placed 500μm-1000μm away from HEK293 cell pellet and cultures were analyzed 48 hours after plating.

***Correlated light – electron microscopy analysis***

Brains dissected from mice (transcardially perfused with 1% glutaraldehyde- 2.5% paraformaldehyde in phosphate buffered saline, and post-fixed overnight at 4°C in the same blocking solution) were affixed ventral side up under the dissection microscope. Excess buffer was blotted from the surface with Whatman paper. Meninges covering pontine grey nuclei were removed. DiI crystals were inserted with a galvanized wire tool approximately 2mm under the surface into both PGNs, ranging from approximately four crystals per nucleus at P0 to approximately 8 crystals at P21. Excess DiI was blotted from brain surface with small pieces of Whatman paper. In all subsequent steps samples were protected from light. Brains were immersed in phosphate buffered saline containing 0.02% sodium azide (PBS-Az) in sealed glass vials, and incubated at 37°C, for P0, 10 days: P7,15 days; P14, 28 days; P21, 60 days. After the appropriate incubation times, brains were either stored at 4°C (maximally for a few weeks) or immediately processed. For photo-oxidation, coronal 50**μ**m vibratome sections containing cerebellar hemispheres were collected in PBS-Az, and either processed immediately, or stored at 4°C for a maximum of 2 days. Sections chosen for photo-oxidation were transferred with a brush into a drop of 0.1M Tris-HCl buffer, pH8.2 on a glass slide. Tris buffer was replaced with 30μl of Diaminobenzidine (DAB) solution (1.5 mg/ml in 0.1M Tris-HCl buffer, pH8.2) prepared on the day of the experiment (Wako Chemical Co.), filtered through a 0.2μl syringe filter, and kept at 4°C protected from light. The desired area was illuminated with a broad excitation filter (500-550 nm). Illumination times were 12-15 minutes on a 20X Nikon Neofluor (NA=0.75) objective, or 22-28 minutes with a 10X Neofluor (NA=0.5) objective and a 100W mercury lamp on a Nikon Optiphot microscope. The progression of the color reaction was carefully monitored by transmission light microscopy and stopped when mossy fibers appeared sufficiently dark (orange-brown). Sections were rinsed several times in PBS, and post-fixed for 10 minutes in 2% glutaraldehyde in PBS.

For calbindin double-labeling, photo-oxidized sections were rinsed with PBS, then blocked in 5% bovine serum albumin (BSA), 0.5% Triton X-100 (Tx100) in PBS for 1 hour. Sections were incubated with rabbit anti-calbindin antibodies, diluted 1:5,000 in the same blocking solution for 4 days, rocking at 4°C. After six 10 minute washes in PBS, sections were incubated in 1:500 anti-rabbit horseradish peroxidase for 36-48 hours, rocking at 4°C followed by six 10 min washes in PBS. Sections were developed in 0.5 mg/ml DAB, 0.05M Tris-HCl, pH7.6, 0.001% hydrogen peroxide. Sections were rinsed in PBS several times, and post-fixed for 10 minutes in 2% glutaraldehyde in PBS.

All sections further processed for electron microscopy were fixed in 1% osmium tetroxide, rinsed, dehydrated in a series of ethanols, and infiltrated with Epox in a Pelco EM microwave. Sections were embedded in Epox between two plastic slides, and allowed to polymerize for 24-48 hours at 60°C. Epox wafers containing embedded sections were separated, taped down on glass slides and viewed by transmitted light, DIC, and phase optics. Areas of interest from the 50µm sections containing mossy fiber axon arbors in the PCL were drawn with the aid of Leitz *camera lucida* and 100x oil immersion objective.

Areas of interest selected for EM were drawn, then cut out, mounted on a blank Epon stub, and sectioned at 7μm with a Diatome HistoKnife. The 7μm semithin sections were placed on glass slides and coverslipped in immersion oil, and the areas of interest were re-drawn and photographed with phase optics using a 63x oil immersion objective. By comparing drawings of semithin and thick sections, presumed contacts and their position on the axon arbor were identified. Semithin sections of interest were remounted on faced-off Epon stubs, trimmed, thin sectioned at approximately 70 nm, and collected serially on single-holed Formvar-coated grids. Grids were stained with uranyl acetate and lead citrate, and examined with a JEOL 1200EX electron microscope. The drawings and the photographs of the thick and semithin sections served as roadmaps for relocating specific terminals. On average, 100 serial sections encompassing a 7μm thick region were examined per putative contact.

**Supplemental References**

Baird, D.H., Baptista, C.A., Wang, L.C., and Mason, C.A. (1992). Specificity of a target cell-derived stop signal for afferent axonal growth. Journal of neurobiology *23*, 579-591.

Chang, W., Lin, Z., Kulessa, H., Hebert, J., Hogan, B.L., and Wu, D.K. (2008). Bmp4 is essential for the formation of the vestibular apparatus that detects angular head movements. PLoS Genet *4*, e1000050.

Saito, H., Tsumura, H., Otake, S., Nishida, A., Furukawa, T., and Suzuki, N. (2005). L7/Pcp-2-specific expression of Cre recombinase using knock-in approach. Biochem Biophys Res Commun *331*, 1216-1221.

Schaeren-Wiemers, N., and Gerfin-Moser, A. (1993). A single protocol to detect transcripts of various types and expression levels in neural tissue and cultured cells: in situ hybridization using digoxigenin-labelled cRNA probes. Histochemistry *100*, 431-440.

Srinivas, S., Watanabe, T., Lin, C.S., William, C.M., Tanabe, Y., Jessell, T.M., and Costantini, F. (2001). Cre reporter strains produced by targeted insertion of EYFP and ECFP into the ROSA26 locus. BMC Dev Biol *1*, 4.

Zhang, Q., and Mason, C.A. (1998). Developmental regulation of mossy fiber afferent interactions with target granule cells. Dev Biol *195*, 75-87.
