# Supplementary figures and images for: Development of Axon-Target Specificity of Ponto-Cerebellar Afferents
Source: PLoS Biol. 2011 Feb 8;9(2):e1001013. doi: 10.1371/journal.pbio.1001013 (PMC3035609; doi:10.1371/journal.pbio.1001013)

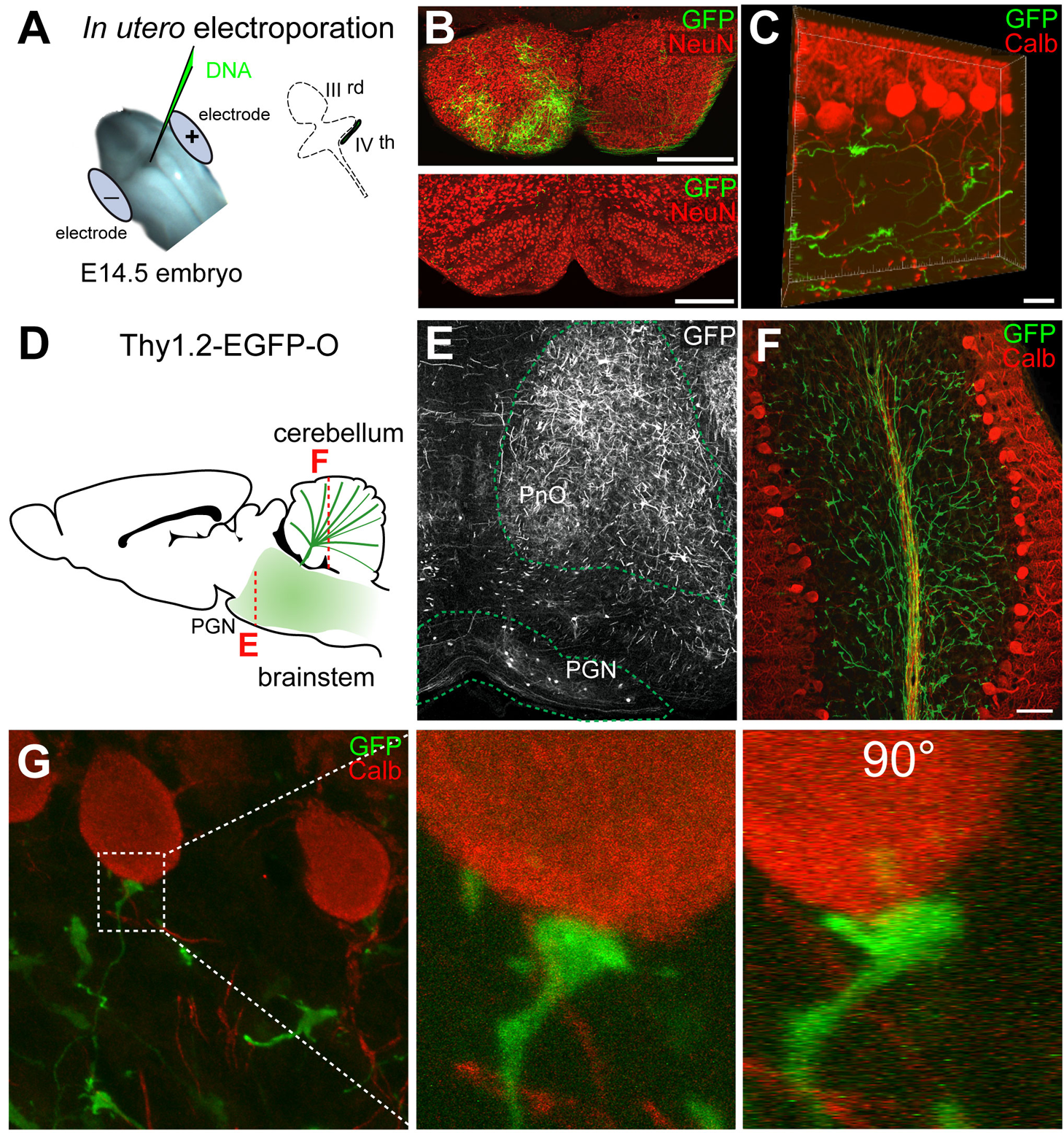

Supplement: Figure S1 — Marking of mossy fiber afferents by in utero electroporation and in transgenic mice. (A) Dorsal view of an E14.5 mouse embryo and schematic drawing of the neural tube at this stage. The fourth ventricle, the site of DNA injection for the in utero electroporation, is marked. DNA is delivered unilaterally into precursor cells flanking one side of the ventricle. (B) Coronal sections of pontine gray nucleus and inferior olivary nucleus from electroporated mice (P7) demonstrating selective marking of pontine gray nucleus neurons and absence of labeling in the inferior olive. (C) Pontine mossy fiber axons marked by EGFP expression (green) introduced by in utero electroporation and analyzed at P21. Purkinje cells are marked with antibodies to calbindin (red). (D) Schematic diagram illustrating widespread GFP-positive brainstem areas (green shading) in GFP-O transgenic mice at postnatal day 14. Red dashed lines indicate planes of coronal tissue sections shown in (E) and (F). (E) GFP fluorescence in a coronal section through P14 brainstem is observed in few cell bodies located in the Pontine Gray Nucleus (PGN) and in a significant number of cell bodies of the Oral Pontine Reticular Nucleus (PnO) amongst other brainstem nuclei (not shown). (F) Coronal section through the cerebellar hemisphere (P21) showing calbindin-labeled Purkinje cells (red) and GFP-labeled fibers (green) with typical mossy fiber morphology in the WM and the IGL. Labeling in the cerebellar hemispheres of GFP-O mice is mostly restricted to mossy fibers. (G) Direct contacts between mossy fibers (green) and Purkinje cell soma (anti-calbindin, red) and axons (P14). The middle panel shows an enlargement of a contact. The right panel represents a projection of the confocal image stack rotated by 90° confirming the juxtaposition of mossy fiber ending and Purkinje cell soma. Scale bars: (B) upper panel 500 µm, lower panel 200 µm; (C) 20 µm; (F) 100 µm. (TIF) [file pbio.1001013.s002.tif]

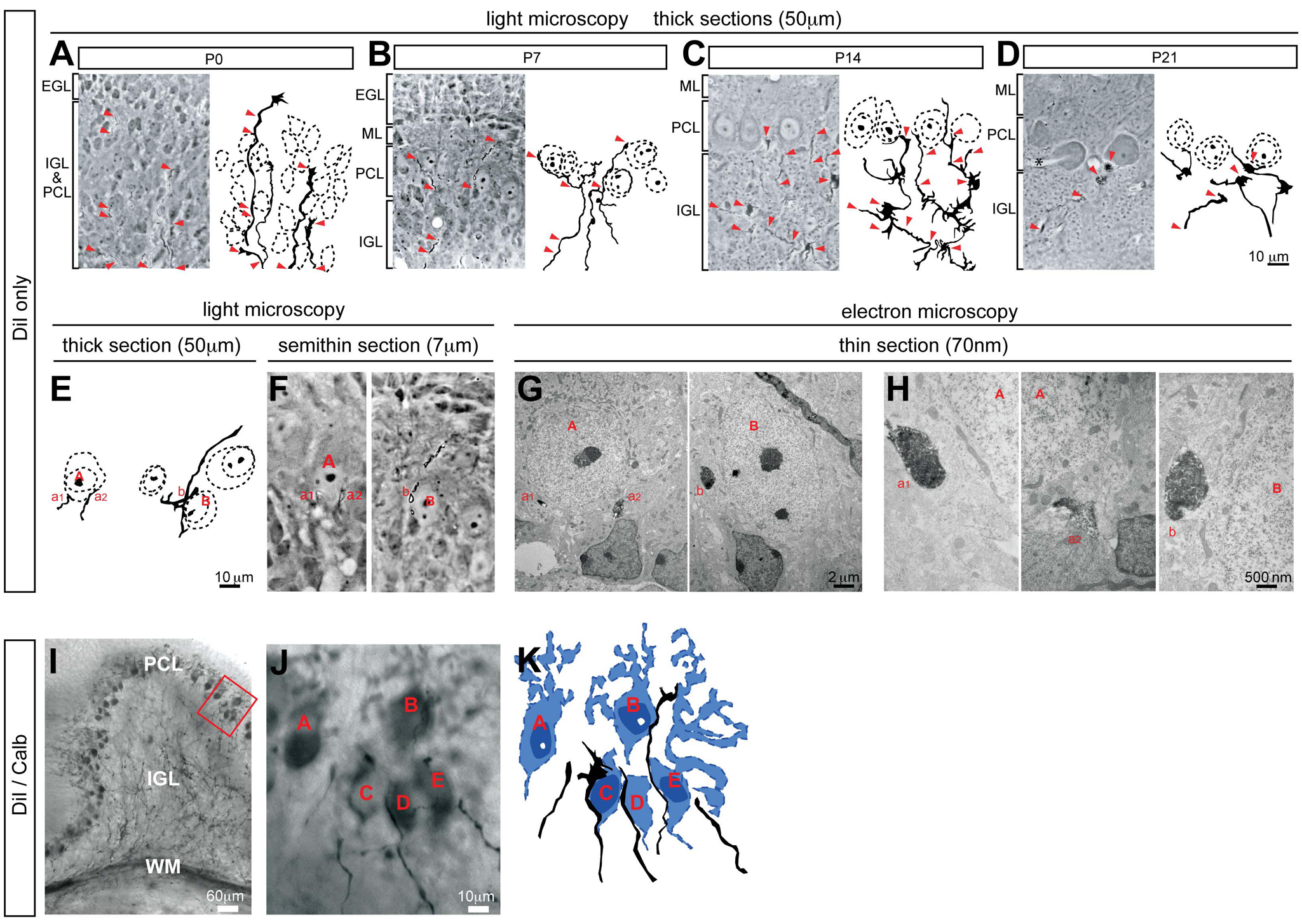

Supplement: Figure S2 — Correlated light and electron microscopic analysis. (A–D) Photographs and camera lucida tracings of the IGL and PCL in DiI labeled 50 µm thick sections from P0, P7, P14, and P21 time points. In the photographs a single plane of focus is seen, such that labeled mossy fibers appear as discontinuous segments (demarcated by red arrowheads). Somata of Purkinje and granule cells can be identified by DIC microscopy. The two cell types can be distinguished by unique cytological profiles (soma diameter, nucleoli, density of cytoplasm and nuclei). Corresponding camera lucida drawings, reconstructing mossy fiber arbors through the Z-planes of the 50 µm thick sections, are shown to the right side of the photographs (red arrowheads demarcate the same mossy fiber segments as in the photographs). Black asterisk indicates blood vessel. Scale bar: 10 µm. (E–H) Analysis steps in correlated light-electron microscopy. (E) Putative contacts (a1, a2, and b) between pontine mossy fibers and Purkinje cell somata identified from camera lucida tracings of 50 µm thick section. (F) Photograph of 7 µm semithin section of corresponding area verifying that putative contacts a1, a2, and b are indeed immediately adjacent to the soma of Purkinje cells A and B. (G) Electron micrographs of 70 nm thin sections corresponding to the same area as in (E) and (F). Cellular landmarks, such as blood vessels, or Purkinje and granule cell nuclei and nucleoli, allow correlation of cellular features and relationships identified at light level with the ultrastructural profiles. (H) Electron micrographs of contacts a1, a2, and b taken at 30,000× magnification showing that these are direct cellular appositions between mossy fibers and Purkinje cell somata (a1 and b) and on perisomatic protrusions (a2), respectively. All three contacts show ultrastructural features of synapses (polarization of synaptic vesicles and PSDs). (I,J,K) DiI-calbindin double-labeled sections. Calbindin immunohistochemistry facilitates identi [file pbio.1001013.s003.tif]

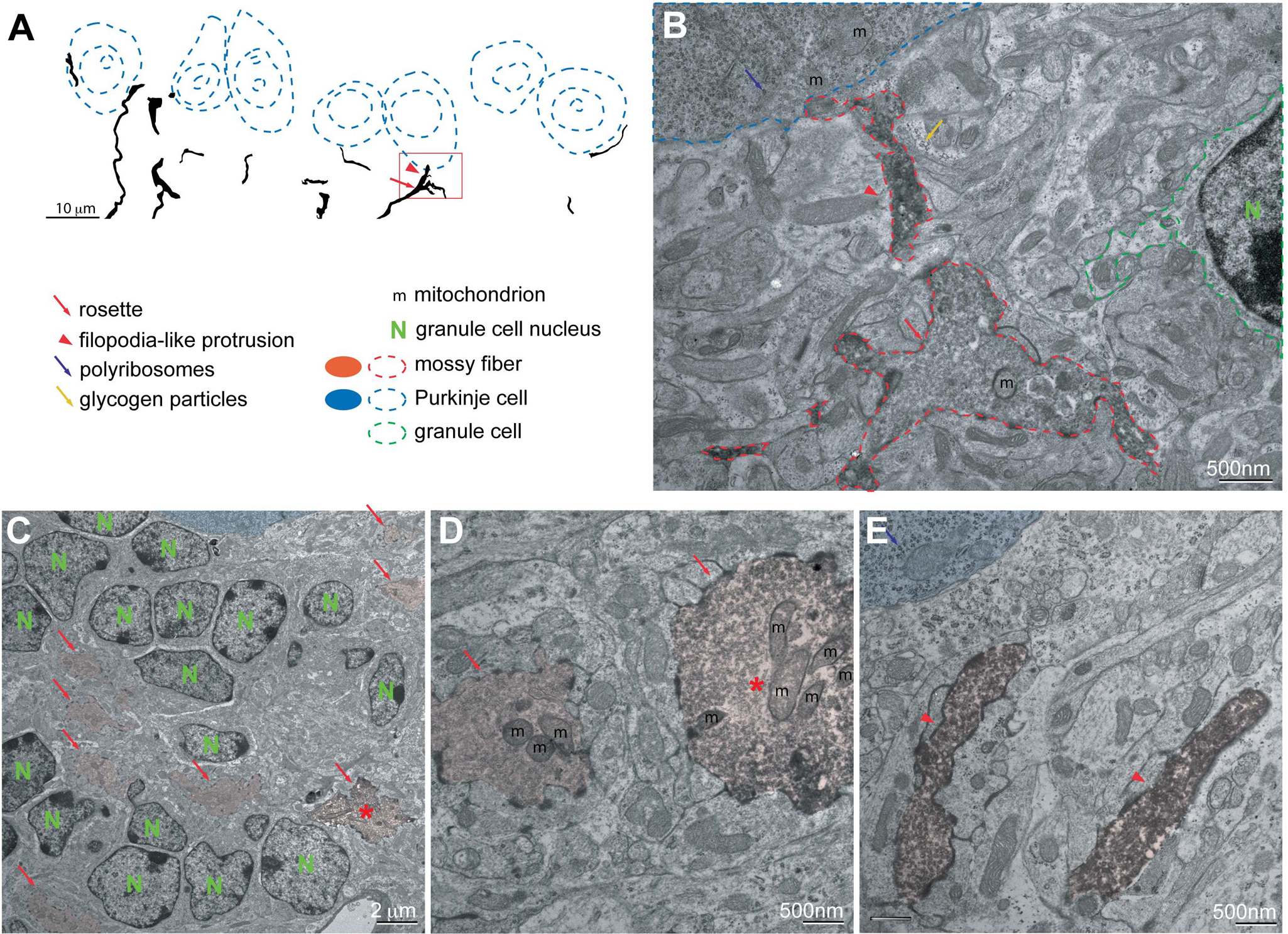

Supplement: Figure S3 — The cytological features used for unambiguous identification of Purkinje cells and mossy fiber processes in the correlated light–EM analysis. (A) Camera lucida tracing showing mossy fiber segments (black) and outlines of Purkinje cell somata, nuclei, and nucleoli (blue dotted lines) identified by DIC microscopy. Drawings obtain with a camera lucida and 100× objective in DiI-labeled 50 µm thick section from P14 cerebellum. Area boxed in red contains a rosette (highlighted by red arrow), a filopodia-like protrusion (red arrowhead), and establishes a putative contact with the soma of a Purkinje cell. Scale bar: 10 µm. (B) Electron micrograph of a 70 nm thin section corresponding to the boxed area in (A). The labeled mossy fiber including the portion of the rosette is outlined by a dashed orange line, and a Purkinje cell soma is outlined in blue. Cerebellar cell types can be distinguished in electron micrographs by unique ultrastructural features: clusters of polyribosomes (blue arrow) in the Purkinje cell somatic cytoplasm; dark, densely packed chromatin (green N) distinguishes granule cell nuclei; very light cytoplasm and glycogen particles (yellow arrow) distinguish glial cell processes. Scale bar: 2 µm. (C) Electron micrograph of the IGL/PCL boundary containing seven unlabeled and one labeled (asterisk) mossy fiber rosettes highlighted by orange overlay. Mossy fiber rosettes are distinguished by their large diameter, irregular outline, and abundance of synaptic vesicles, mitochondria, and multiple active zones juxtaposed to PSDs on the surrounding processes. Scale bar: 2 µm. (D) Higher magnification of an unlabeled (left) and labeled (right, asterisk) mossy fiber rosette (orange overlay and red arrows). Even though all the lipid membranes appear darker due to incorporation of DiI, the ultrastructural characteristics are identical to unlabeled rosettes. Scale bar: 500 nm. (E) DiI-labeled thin mossy fiber processes in the upper IGL, near but not in contact with the Pu [file pbio.1001013.s004.tif]

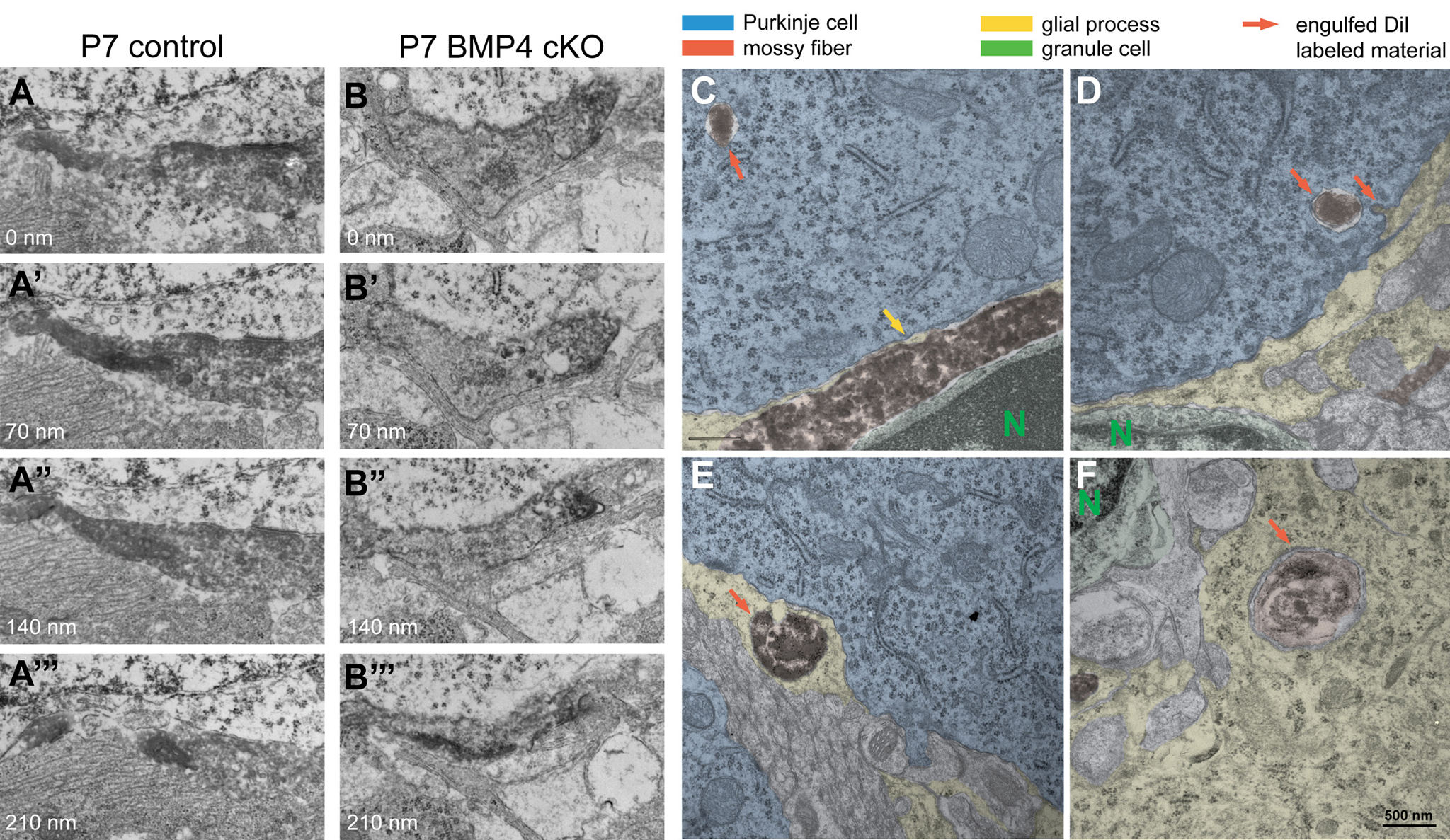

Supplement: Figure S4 — Ultrastructural features of mossy fiber–Purkinje cell interactions. (A) Serial sections of the mossy fiber–Purkinje cell synaptic contact in wild-type cerebellum shown in Figure 3B (P7). (B) Serial sections of the mossy fiber–Purkinje cell contact shown in Figure 7E in BMP4 cKO mouse cerebellum (P7). Note that pre- and postsynaptic densities are often lacking, the membrane between the mossy fiber and Purkinje cell soma is ruffled, and synaptic vesicles are irregularly shaped. (C–D) Electron micrograph (P14) showing engulfment of DiI-labeled mossy fiber process in Purkinje cell (blue overlay, red arrows, one revealing an endocytotic figure nearby). Thin glial processes can frequently be seen separating mossy fibers from the Purkinje cell soma (yellow arrow, C). (E–F) Glial ensheathment of mossy fiber processes near Purkinje cell soma in P14 wild-type cerebellum. Glial processes (identified by the presence of glycogen particles in the cytoplasm, yellow overlay) can frequently be seen to separate mossy processes from the Purkinje cell soma. Scale bar: 500 nm; “N” marks granule cell nuclei (green). (TIF) [file pbio.1001013.s005.tif]

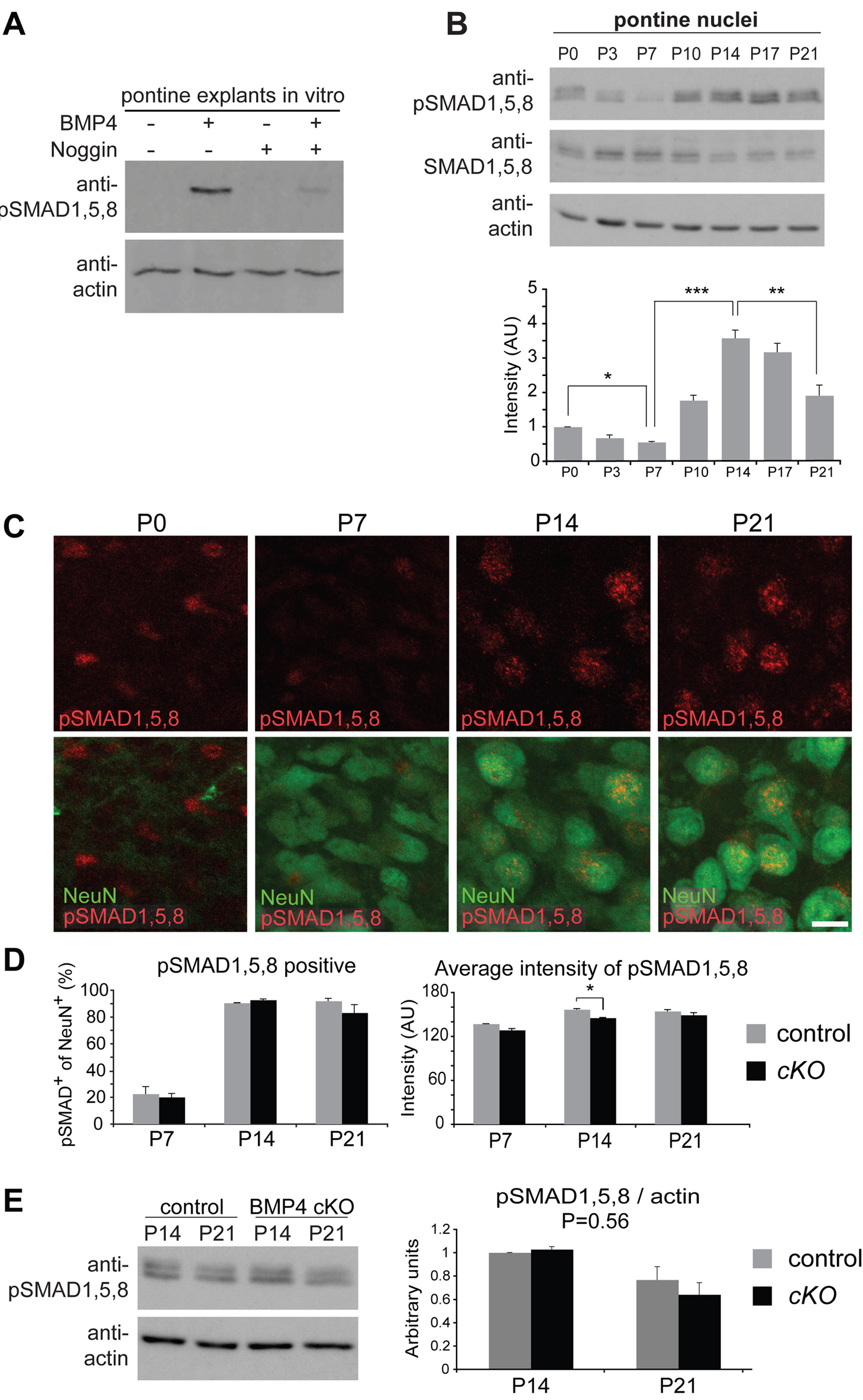

Supplement: Figure S5 — Developmental analysis of SMAD phosphorylation in pontine gray nuclei. (A) Activation of the BMP signaling pathway in pontine explants cultured in vitro. Western blots for the phosphorylated forms of SMAD1, 5, and 8 (pSMAD1,5,8) in PGN explants (2 d in vitro), treated with recombinant BMP4 (10 ng/ml) and/or noggin (100 ng/ml). (B) Western blots for SMAD1, 5, and 8 (SMAD1,5,8), their phosphorylated forms (pSMAD1,5,8), and actin in the PGN at P0, P3, P7, P10, P14, P17, and P21, showing dynamic changes in the levels of pSMAD1,5,8 in the PGN during development. The graph shows a quantitative assessment of SMAD1,5,8 activation during development measured by Western blot and expressed as a ratio of pSMAD1,5,8 to actin immune-reactivity in the cell extracts. The intensity ratio obtained for the P0 time point was set to “one” and measurements for other developmental time points are expressed relative to P0. (C) Immunohistochemistry for pSMAD1,5,8 (red) and NeuN (green) detected in parasagittal sections through the PGN at P0, P7, P14, and P21. Note the increase in number and intensity of pSMAD1,5,8 nuclear labeling at P14 and P21 compared to P7. Scale bar: 10 µm. (D) Comparison of SMAD1,5,8 activation in control (BMP4+/+::Pcp2cre/cre) and cKO (BMP4fl/fl::Pcp2cre/cre) at P7, P14, and P21 in the PGN assessed by immunohistochemistry. PGN sections were immunolabelled for pSMAD1,5,8 and NeuN (a marker of pontine projection neurons). Left graph shows the percentage of pSMAD1,5,8-positive cells amongst the NeuN-positive neurons and right graph shows the average intensity of the nuclear pSMAD1,5,8 labeling (poor NeuN immune-reactivity at P0 precluded quantitative analysis of this earliest time point). Gray bars are control, black bars are cKO. * p<0.05, ** p<0.01, *** p<0.001. (E) Western blot analysis of pSMAD1,5,8 levels in pontine nuclei at P14 and P21 reveals no significant differences in BMP4+/+::Pcp2cre/cre (control) and BMP4fl/fl::Pcp2cre/cre (BMP4 cKO) mice. pSMAD levels wer [file pbio.1001013.s006.tif]

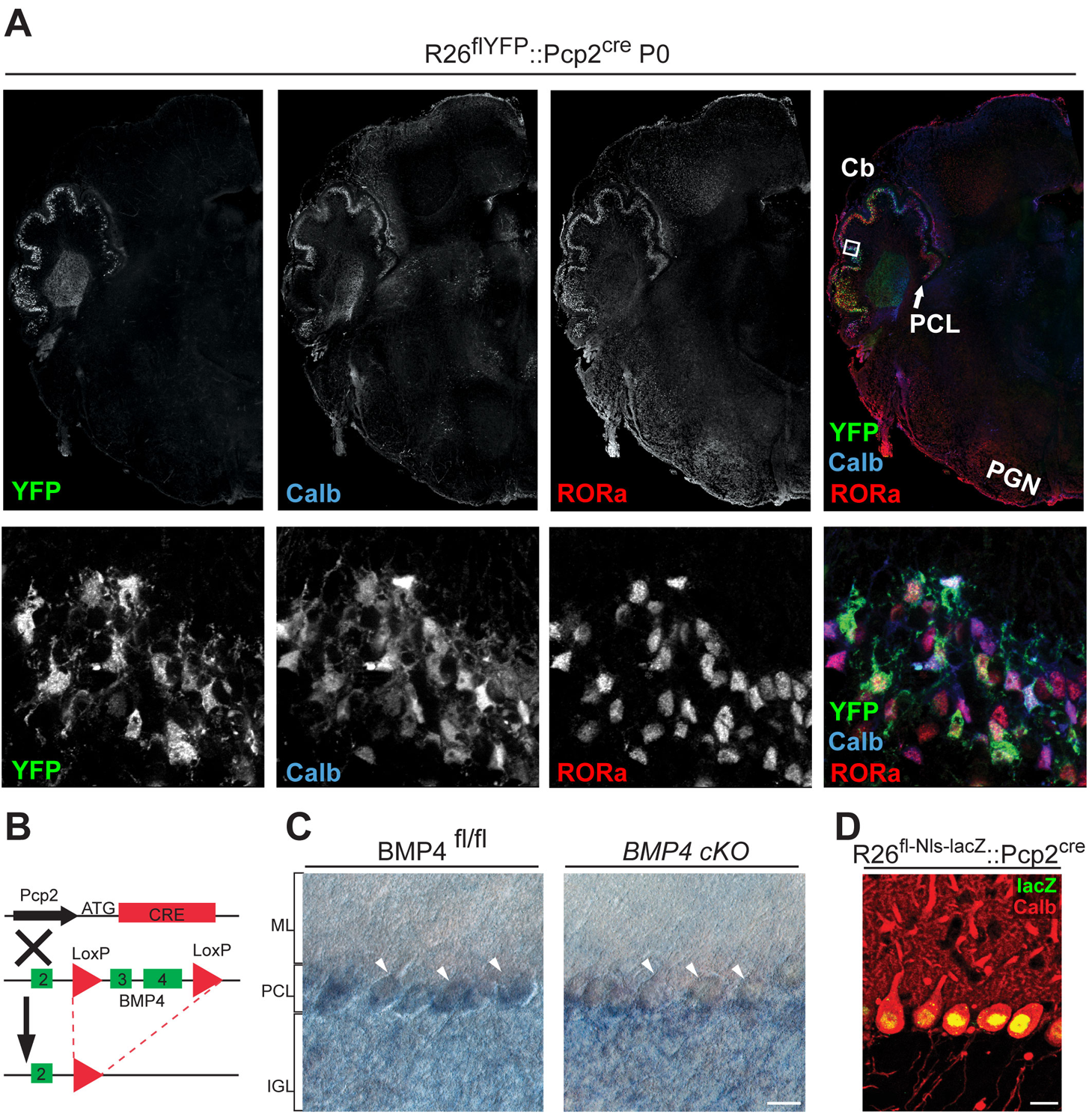

Supplement: Figure S6 — Conditional ablation of BMP4 using Pcp2cre KI mice. BMP4 conditional KO mice were obtained by crossing a floxed BMP4 allele [81] and Pcp2 cre knock-in mice [54] which express cre recombinase from the Pcp2 locus. (A) The cell-type specificity of cre-mediated recombination in Pcp2-cre knock-in mice was confirmed using a ROSA26-lox-stop-lox-YFP reporter [83]. Recombination is observed specifically in Purkinje cells (co-labeled with calbindin which labels a subset and RORalpha which labels all Purkinje cells) but not in the brain stem. Note that at P0 recombination in Purkinje cells throughout the cerebellum is detected in coronal sections. Higher magnification views (lower row) reveal that at least 50% of RORalpha positive Purkinje cells show activation of the reporter. (B) Schematic drawing of the conditional BMP4 locus. Exons 3 and 4 are flanked by loxP sites. (C) Confirmation of BMP4 expression in Purkinje cells (P14) by in situ hybridization with a probe for the ablated exon 3. Individual Purkinje cells are marked by arrowheads. Note that the high sequence similarity between BMP4 exon 3 and other BMP family members makes detection more challenging than in the experiments using probes against the UTR used in Figure 4. (D) Visualization of cre-mediated recombination in Purkinje cells at P14 using a ROSA26-lox-stop-lox-nuclear lacZ reporter allele. Purkinje cells are labeled with anti-calbindin antibodies. Scale bars: (C) 30 µm; (D) 20 µm. (TIF) [file pbio.1001013.s007.tif]

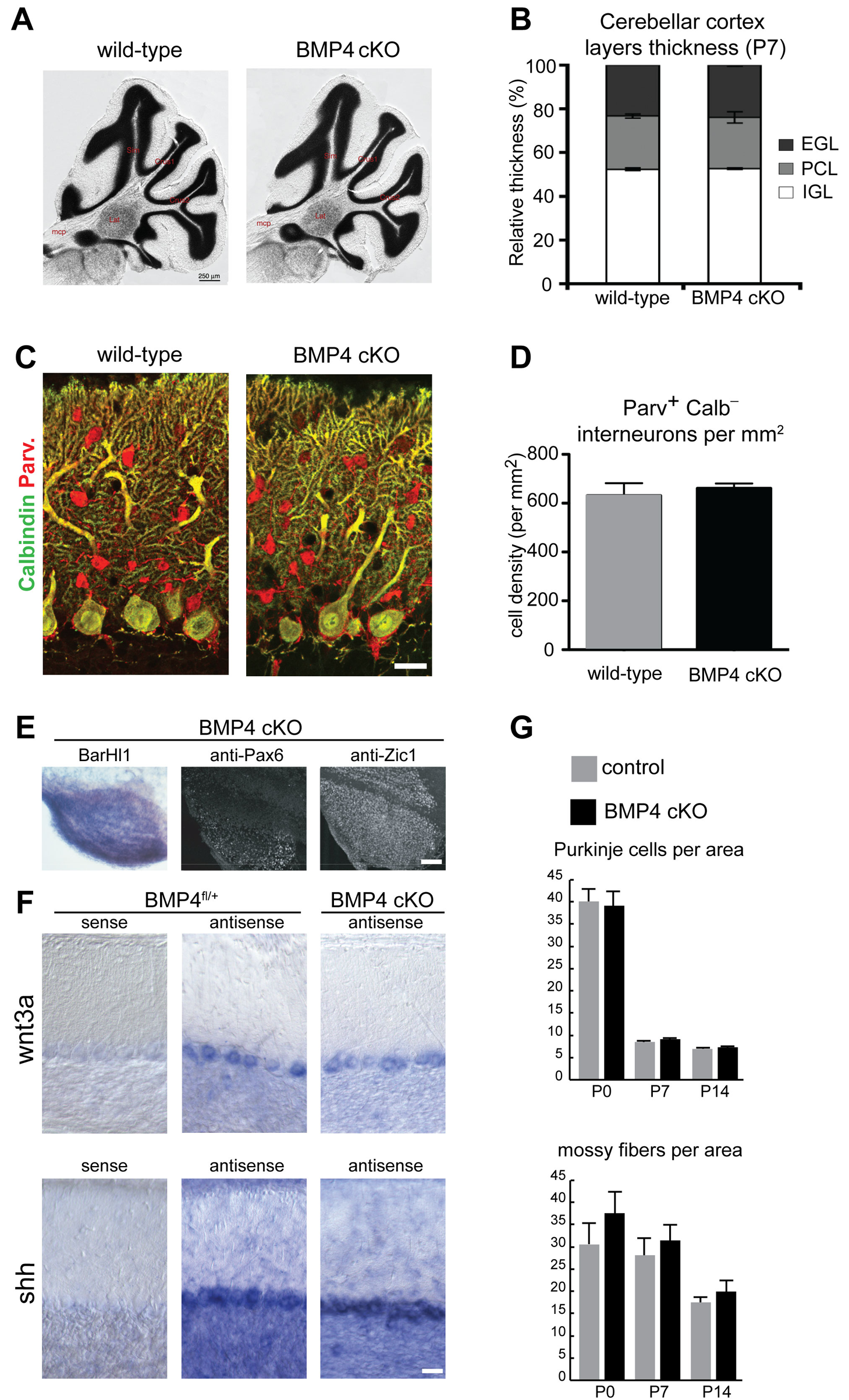

Supplement: Figure S7 — Characterization of BMP4 cKO cerebellum. (A) Normal morphology and layering of the cerebellum in P21 wild-type and BMP4 cKO mice analyzed by Nissl staining. Representative sections of the mediolateral cerebellum are shown. Scale bar: 250 µm. (B) No significant alterations in the thickness of EGL, IGL, and WM in wild-type and BMP4 cKO mice. Thickness of layers was measured in Hoechst-stained parasagittal sections from P7 animals and relative thickness of external germinal layer (EGL), Purkinje cell layer (PCL), and internal granular layer (IGL) was measured (n=8 sections from 2 animals for each genotype). (C) Stellate and basket cell interneurons were identified as parvalbumin (red)-positive and calbindin (green)-negative cells. The density and morphology was not noticeably altered in cerebella of BMP4 cKO mice as compared to wild-type. (D) Cell densities of parvalbumin-positive/calbindin-negative cells (Parv+Calb−) were quantified from 6–7 fields per genotype from wild-type and BMP4 cKO animals. Cell numbers per area of molecular layer (in mm2) are compared. (E) The pontine projection neuron markers Bar-Homologue-like 1 (BarHl1), Pax6, and Zic1 are expressed in pontine nuclei from mutant mice. Images show in situ hybridization (BarHl1) and immunohistochemistry (anti-Pax6 and anti-Zic1) in BMP4 conditional mutant mice at P0 time point. Scale bar: 100 µm. (F) Expression of wnt3a and shh mRNAs in heterozygous (BMP4fl/+::Pcp2cre/+) and homozygous (BMP4fl/fl::Pcp2cre/+) conditional mutant cerebella (P14) detected by in situ hybridization. Sense control probes are shown on the left. Scale bar: 20 µm. (G) Quantitative analysis of mossy fiber and Purkinje cell numbers from camera lucida drawings (encompassing all of the mossy fiber segments and Purkinje cell outlines reconstructed from an area (175 µm×120 µm×30 µm), oriented parallel to the PCL, and recorded with a 100× objective) of P0, P7, and P14 time points from control littermates (BMP4fl/fl) and BMP4 cKO animals (>15 [file pbio.1001013.s008.tif]

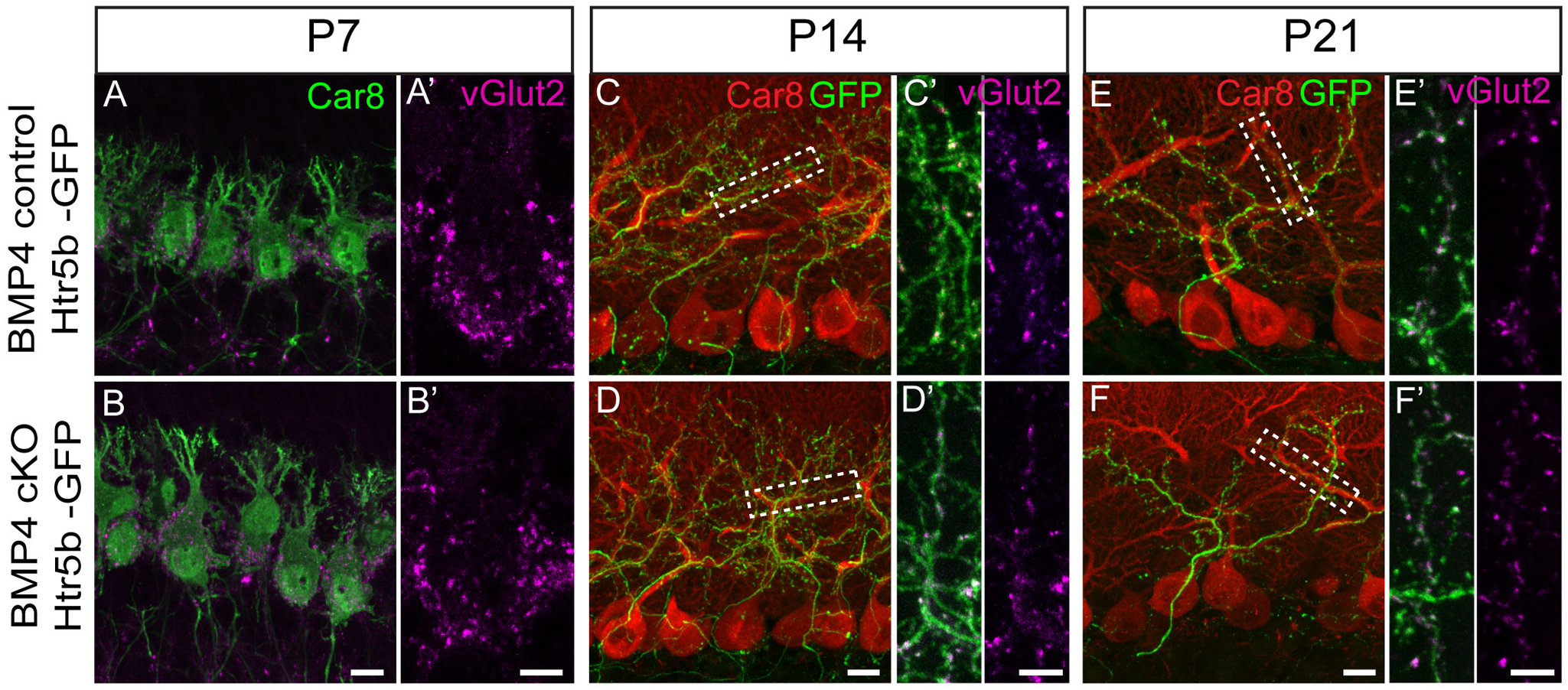

Supplement: Figure S8 — Climbing fiber morphology is not significantly altered in BMP4 cKO mice. The BAC transgenic line Htr5b-GFP shows expression of GFP in a subset of inferior olivary neurons starting at P14 and was used to examine climbing fiber morphology. Sagittal sections of P7 (A, B), P14 (C, D), and P21 (E, F) of control (upper panels, BMP4+l+::Pcp2cre/cre) and BMP4 cKO (lower panels, BMP4fllfl::Pcp2cre/cre) cerebellar cortex. (A′, B′, C′, D′, E′, F′) show enlargements and demonstrate similar climbing fiber (anti-GFP, green in C, D, E and F) morphology, presynaptic synaptic vesicle accumulation (anti-vGlut2, magenta), and arborization along the Purkinje cell dendrites (anti-Car8, green in A, B, and red in C, D, E, F) in control and mutant cerebella. Scale bars: (B, D, F) 10 µm; (B′, D′, F′) 5 µm. (TIF) [file pbio.1001013.s009.tif]
